# Alkaloid diversity expansion of a talent fungus *Penicillium raistrichii* through OSMAC-based cultivation

**DOI:** 10.3389/fmicb.2023.1279140

**Published:** 2023-11-09

**Authors:** Meijia Zhong, Huihui Kang, Weizhong Liu, Liying Ma, Desheng Liu

**Affiliations:** Laboratory of Natural Drug Discovery and Research, College of Pharmacy, Binzhou Medical University, Yantai, China

**Keywords:** alkaloidal secondary metabolites, antimicrobial activity, *β*-carboline, OSMAC strategy, *Penicillium raistrichii*, 2-quinolinone

## Abstract

**Introduction:**

Alkaloidal natural products are attractive for their broad spectrum of pharmaceutical bioactivities. In the present work, the highly productive saline soil derived fungus, *Penicillium raistrichii*, was subjected to the strategy of OSMAC (one strain many compounds) with changes of cultivation status. Then, the work-flow led to the expansion of the alkaloid chemical diversity and subsequently induced the accumulation of four undescribed alkaloids, named raistrimides A–D (**1–4**), including three *β*-carbolines (**1–3**), one 2-quinolinone (**4**), and one new natural product, 2-quinolinone (**5**), along with five known alkaloid chemicals (**6–10**).

**Methods:**

A set of NMR techniques including ^1^H, ^13^C, HSQC and HMBC, along with other spectroscopic data of UV-Vis, IR and HRESIMS, were introduced to assign the plain structures of compounds **1–10**. The absolute configuration of **1–3** were elucidated by means of X-ray crystallography or spectroscopic analyses on optical rotation values and experimental electronic circular dichroism (ECD) data. In addition, it was the first report on the confirmation of structures of **6**, **7** and **9** by X-ray crystallography data. The micro-broth dilution method was applied to evaluate antimicrobial effect of all compounds towards *Staphylococcus aureus*, *Escherichia coli,* and *Candida albicans*.

**Results and discussion:**

The results indicated compounds **1**, **3** and **4** to be bioactive, which may be potential for further development of anti-antimicrobial agents. The finding in this work implied that OSMAC strategy was a powerful and effective tool for promotion of new chemical entities from *P. raistrichii*.

## Introduction

It is clearly understood that the secondary metabolite (SM) production capacity of microbes lies on biosynthetic gene clusters (BGCs) (Bills and Gloer, 2016; Genilloud, 2019; Zhang J. J. et al., 2019; Ke and Yoshikuni, 2020; Alexander, 2023). Yet, the traditional workflow involving laboratory cultivation of microbes, followed by extraction of fermentation, SMs purification, and structure elucidation, often results in the silent or weak expression of BGCs and thus confines the occurrences and number of the new chemical entities, due to failure of resembling the nature environment with intense microbial competition. There is a growing consensus that unlocking the silent BGCs is a key channel to counteract the dramatic decline in the quantity of novel naturally-produced chemical structures (Reen et al., 2015; Zhang X. et al., 2019).

During the recent several decades, in order to tap the biosynthetic potential and strengthen silent gene expression, molecular methodologies (Reen et al., 2015; Palys et al., 2020; Wang et al., 2020; Han et al., 2020a,b) like gene manipulation, including knocking down, introduction or heterologous expression, regulation of promoters, and induction of mutations, as well as approaches based on cultivation conditions variation, have been employed and have achieved great successes in mining cryptic SMs. Of these, the one strain many compounds (OSMAC) strategy, covering medium variation, cultivation condition change, co-cultivation, or addition of epigenetic modifiers, has been efficiently applied to promote new therapeutic agents discovery, highlighting it as an easy and productive approach to trigger the reaction of the crypt BGCs and enhance the natural SMs production according to the extensive literature reports (Bode et al., 2002; Romano et al., 2018; Pan et al., 2019).

**Figure 1:**
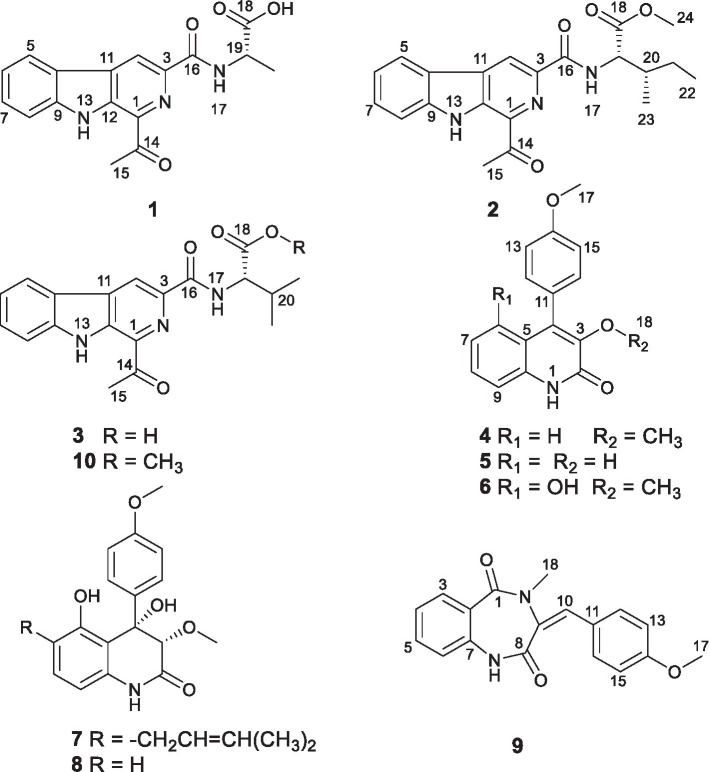
Chemical structures of compounds **1–10**.

During our ongoing efforts in pursuit of therapeutic pharmaceuticals from saline soil-derived fungi, a talent genus *P. raistrichii* isolated from saline sediment in Bohai Bay, entered into our vision. Chemical investigations on the metabolic profile resulted in the discovery of several different novel scaffolds including polyketides and alkaloids with biological behaviors of antiinsectan, antimicrobial, radical scavenging, cytotoxic, or anti-HCV activities (Belofsky et al., 1998; Ma et al., 2016; Jia et al., 2017; Liu et al., 2018; Li et al., 2019). In recognition of the superior ability for the production of intriguing molecules from the talent strain, and also enlightened by OSMAC strategy of expansion on the fungal metabolome, the strain was subjected to culture under static conditions in a liquid medium. Excitingly, the change of culture conditions, which may have exerted influences on the biochemical reactions, caused the strain to produce four new alkaloids, named raistrimides A-D (**1**–**4**), one new natural product 2-quinolinone, 3-hydroxy-4-(4′-methoxyphenyl)-2(1*H*)-quinolinone (**5**) (He et al., 2015), and five known alkaloids, pesimquinolone R (**6**) (Dai et al., 2021), peniprequinolone (**7**) (Hayashi et al., 1997; Uchida et al., 2006), 4,5-dihydroxy-3,4-dihydro-3-methoxy-4-(4′-methoxyphenyl)-2(1*H*)-quinolinone (**8**) (Framm et al., 1973), dehydrocyclopeptin (**9**) (Ishikawa et al., 2014), and tunicoidine *F* (**10**) (Wen et al., 2014; Zhang et al., 2015). Detailed information about the isolation, purification, and structure elucidation, as well as the antimicrobial effect assessment of these compounds (Figure 1) is presented in this work.

## Materials and methods

### General experimental procedures

An uncorrected XRC-1 micro melting point apparatus was used to measure the melting points. Uv–Vis and IR data were performed on a TU-1091 spectrophotometer and a Thermo Nicolet 6,700 infrared spectrophotometer, respectively. A Rudolph Autopol V Plus digital polarimeter, a Bruker Smart 1,000 CCD X-ray diffractometer, and an Applied Photophysics Chirascan spectropolarimeter were used for the determination of optical rotations, X-ray crystal data, and electron circular dichroism (ECD) spectra, respectively. Varian Inova 600 and Bruker AV-400/AVIII 500 spectrometers were used to measure 1D and 2D NMR data in different deuterium solvents using tetramethylsilane as an internal standard (IS). HRESIMS spectra were recorded using an Agilent 1200RRLC-6520 Accurate-Mass Q-TOF LC/MS mass spectrometer or a Waters Q-TOF Ultima GLOBAL GAA076 LC. A Shimadzu LC-6 AD and LC-20A Liquid Chromatography were applied for the preparative isolation and analysis on HyperClone C_18_ columns (5 μm, 10 × 250 mm, 5 μm, 4.6 × 250 mm, respectively). Open column chromatography (CC) isolations were performed on silica gel (200–300 mesh, Qingdao Marine Chemical, China), reverse phase (RP) C_18_ material (Pharmacia Fine Chemical Co., Sweden), and Sephadex LH-20 (Ge Healthcare Bio-Sciences AB, Sweden).

### Fungal material

Saline soil samples were collected from the coast in the Circum-Bohai-Sea region in Zhanhua county, from which a talent strain of *Penicillium raistrickii* with GenBank Accession NO. HQ717799 was isolated in August 2008. The voucher specimen was preserved at the laboratory of Natural Drug Discovery and Research, Binzhou Medical University.

### Cultivation and extraction

The spores of *P. raistrickii* growing well on potato dextrose agar (PDA) were incubated in 500-mL Erlenmeyer flasks which contained 180 mL of culture medium comprising 2% glucose, 1% maltose, 0.03% yeast extract, 0.003% MgSO_4_·7H_2_O, and 0.005% KH_2_PO_4_ with the natural pH value in seawater gathered from offshore areas near Yantai, and fermented under static condition at about 28°C for 60 days. A whole volume of 40 L culture broth was isolated into supernatant and mycelia through cheesecloth. The supernatant was extracted with ethyl acetate (3 × 30 L), and the mycelia was extracted with methanol (3 × 10 L) to afford crude aqueous extract followed by partitioning with ethyl acetate, which yielded an extract of 45 g.

### Isolation and purification

The whole extract was subjected to a VLC column with silica gel eluting by a step gradient of petroleum ether/CHCl_3_ (from 1:2 to 0:1, v/v) to give five fractions (Fr.s 1–5), and then followed by a step gradient of CHCl_3_/MeOH (from 1:2 to 0:1, v/v) to afford another five fractions (Fr.s 6–10). Among these, Fr. 3 (4.1 g) and Fr. 4 (3.3 g) were separated with MeOH/H_2_O (20:80 to 100:0, v/v) on ODS to afford nine (Fr.s 3.1–3.9) and six (Fr.s 4.1–4.6) subfractions, respectively. Then, compound **1** (9.2 mg) was purified by a Sephadex LH-20 CC from Fr. 4.3 (0.2 g) with MeOH as the eluent. Compounds **2** (3.6 mg) and **10** (16.0 mg) were purified by semipreparative HPLC (MeOH:H_2_O, 70:30, v:v) with a retention time (t*_R_*) of 20.8 min and 17.3 min, respectively. Compound **3** (22.0 mg) was also obtained from semipreparative HPLC (MeOH:H_2_O, 65:35, v:v) with a t*_R_* value of 17.1 min. Compounds **4** (12.6 mg) and **5** (9.3 mg) were given from Fr. 3.8 (0.5 g) which was chromatographed on a silica gel column, eluting with CHCl_3_/MeOH (30:1, v/v). Compounds **6** (14.6 mg) and **8** (32.6 mg) were obtained from Fr. 4.2 (0.7 g) and Fr. 5 (1.7 g) by crystalization in MeOH. Compounds **7** (15.8 mg) and **9** (10.8 mg) were purified by semipreparative HPLC (MeOH:H_2_O, 60:70, v:v) from Fr. 3.3 (0.9 g) and Fr. 4.6 (0.7 g) with values of 26.2 min and 13.8 min, respectively.

#### Raistrimide A (**1**)

Yellowish needle (MeOH); mp 278°C (dec.); UV (MeOH) *λ*_max_ (log *ε*): 377 (3.79), 286 (4.61), 220 (4.56) nm; IR (ATR) *ν*_max_ 3,352, 2,869, 1,728, 1,633, 1,537, 1,493, 1,444, 1,336, 1,195, 1,115, 963, 887, 750, 682 cm^−1^; ^1^H and ^13^C NMR data, see Table 1; HRESIMS (*m/z*): 324.0993 [M – H]^−^ (calcd for C_17_H_14_N_3_O_4_, 324.0990). ${\left[\alpha \right]}_{D}^{20}$ + 10.8 (*c* 0.062, MeOH); ECD (MeOH) *λ*_max_ (Δ*ε*) 229 (− 45.21), 204 (+ 120.47) nm.

**Table 1:** ^1^H and ^13^C NMR data for 1–3 (DMSO-*d*_6_).

NO.	1^a^		2^b^		3^a^	
	*δ*_C_, Type	*δ*_H_, mult (*J* Hz)	*δ*_C_, Type	*δ*_H_, mult (*J* Hz)	*δ*_C_, Type	*δ*_H_, mult (*J* Hz)
1	134.0, C		134.4, C		133.9, C	
3	138.1, C		138.0, C		137.7, C	
4	118.1, CH	9.11, s	118.5, CH	9.11, s	118, CH	9.12, s
5	122.3, CH	8.46, d (7.5)	122.8, CH	8.47, d (7.5)	122.4, CH	8.47, d (7.7)
6	120.9, CH	7.35, t (7.5)	121.3, CH	7.36, t (7.5)	120.9, CH	7.36, t (7.7)
7	129.4, CH	7.64, dd (8.2, 7.5)	129.9, CH	7.64, dd (8.2, 7.5)	129.4, CH	7.65, dd (8.2, 7.7)
8	113.3, CH	7.85, d (8.2)	113.8, CH	7.84, d (8.2)	113.4, CH	7.85, d (8.2)
9	142.4, C		142.8, C		142.4, C	
10	120.3, C		120.7, C		120.3, C	
11	132.0, C		132.5, C		132.1, C	
12	134.9, C		135.4, C		134.9, C	
13		12.23, s		12.26, s		12.26, s
14	200.9, C		200.9, C		200.4, C	
15	25.9, CH_3_	2.93, s	26.2, CH_3_	2.90, s	25.6, CH_3_	2.90, s
16	163.7, C		164.4, C		163.9, C	
17		8.85, d (7.5)		8.65, d (8.3)		8.59, d (8.7)
18	174.0, C		172.3, C		172.8, C	
19	47.9, CH	4.59, m	56.8, CH	4.60, dd (8.3, 5.2)	57.1, CH	4.52, dd (8.7, 4.9)
20	17.7, CH_3_	1.52, d (7.1)	37.2, CH	2.10, m	30.5, CH	2.34, m
21			25.4, CH_2_	1.54, m; 1.31, m	19.2, CH_3_	1.03, d (6.6)
22			16.6, CH_3_	0.95, t (6.7)	17.7, CH_3_	1.03, d (6.6)
23			11.7, CH_3_	0.98, d (7.0)		
24			52.5, CH_3_	3.73, s		
OH		12.89, br s				13.07, s

^a1^H (400 MHz) and ^13^C (100 MHz). ^b1^H (600 MHz) and ^13^C (150 MHz).

#### Raistrimide B (**2**)

yellowish needle (MeOH); mp 196–199°C; UV (MeOH) *λ*_max_ (log *ε*): 377 (3.67), 287 (4.53), 220 (4.44) nm; IR (ATR) *ν*_max_ 3,329, 2,964, 1,740, 1,663, 1,626, 1,515, 1,491, 1,459, 1,204, 1,179, 963, 847, 738, 683 cm^−1^; ^1^H and ^13^C NMR data, see Table 1; HRESIMS (*m/z*): 382.1762 [M + H]^+^ (calcd for C_21_H_24_N_3_O_4_, 382.1761). ${\left[\alpha \right]}_{D}^{20}$ + 4.4 (*c* 0.069, MeOH); ECD (MeOH) *λ*_max_ (Δ*ε*) 280 (+ 0.88), 268 (− 0.02), 240 (+ 0.84), 229 (− 0.50), 204 (+ 3.99) nm.

#### Raistrimide C (**3**)

yellowish needle (MeOH); mp 280°C (dec.); UV (MeOH) *λ*_max_ (log *ε*): 377 (3.73), 286 (4.53), 220 (4.46) nm; IR (ATR) *ν*_max_ 3,394, 2,969, 2,542, 1,745, 1,680, 1,622, 1,542, 1,494, 1,361, 1,302, 1,202, 1,147, 965, 899, 788, 742, 681 cm^−1^; ^1^H and ^13^C NMR data, see Table 1; HRESIMS (*m/z*): 352.1302 [M – H]^−^ (calcd for C_19_H_18_N_3_O_4_, 352.1303). ${\left[\alpha \right]}_{D}^{20}$ + 12.0 (*c* 0.055, MeOH); ECD (MeOH) *λ*_max_ (Δ*ε*) 280 (+ 0.73), 264 (− 0.24), 232 (+ 0.30), 224 (− 0.21), 204 (+ 3.68) nm.

#### Raistrimide D (**4**)

colorless needle (MeOH); mp 230–232°C; UV (MeOH) *λ*_max_ (log *ε*): 337 (3.18), 324 (3.36), 311 (3.35), 298 (3.33), 278 (3.37), 218 (4.03) nm; IR (ATR) *ν*_max_ 1,647, 1,609, 1,553, 1,515, 1,500, 1,434, 1,282, 1,219, 1,175, 1,146, 1,017, 899, 836, 747, 699 cm^−1^; ^1^H and ^13^C NMR data, see Table 2; HRESIMS (*m/z*): 280.0973 [M – H]^−^ (calcd for C_17_H_14_NO_3_, 280.0968).

**Table 2:** ^1^H and ^13^C NMR data for 4 (Py-*d*_5_) and 5 (DMSO-*d*_6_).

NO.	4^a^		5^b^	
	*δ*_C_, Type	*δ*_H_, mult (*J* Hz)	*δ*_C_, Type	*δ*_H_, mult (*J* Hz)
1		13.28, s		12.20, s
2	160.3*, C		158.3, C	
3	147.0, C		142.5, C	
4	138.3, C		121.1, C	
5	121.8, C		125.6, C	
6	127.3, CH	7.39, d (8.0)	126.3, CH	7.33, overlapped
7	122.5, CH	7.15, dd (8.0, 7.5)	122.0, CH	7.12, overlapped
8	129.2, CH	7.48, dd (8.0, 7.5)	124.4, CH	7.12, overlapped
9	115.7, CH	7.60, d (8.0)	115.2, CH	7.33, overlapped
10	137.5, C		133.2, C	
11	126.9, C		123.7, C	
12/16	131.6	7.44, d (8.5)	131.1	7.27, d (8.3)
13/15	114.8	7.18, d (8.5)	113.8	7.07, d (8.3)
14	160.2*, C		158.7, C	
17	55.7, CH_3_	3.78, s	55.1, CH_3_	3.83, s
18	60.3, CH_3_	4.05, s		
OH				9.12, s

^a1^H (500 MHz) and ^13^C (125 MHz). ^b1^H (400 MHz) and ^13^C (100 MHz). ^*^Assignments interchangeable.

#### 3-hydroxy-4-(4′-methoxyphenyl)-2(1*H*)-quinolinone (**5**)

Colorless blocks (MeOH); UV (MeOH) *λ*_max_ (log *ε*): 331 (3.51), 318 (3.65), 306 (3.60), 286 (3.58), 243 (3.86), 222 (4.16) nm; IR (ATR) *ν*_max_ 2,918, 1,652, 1,608, 1,574, 1,515, 1,501, 1,455, 1,403, 1,291, 1,245, 1,031, 950, 881, 753, 713 cm^−1^; ^1^H and ^13^C NMR data, see Table 2; HRESIMS (*m/z*): 266.0815 [M – H]^−^ (calcd for C_16_H_12_NO_3_, 266.0812).

#### Dehydrocyclopeptin (**9**)

colorless blocks (MeOH); 97–99°C; UV (MeOH) *λ*_max_ (log *ε*): 288 (4.28), 214 (4.59), 220 (4.46), 205 (4.43) nm; IR (ATR) *ν*_max_ 3,064, 1,670, 1,622, 1,602, 1,512, 1,478, 1,385, 1,252, 1,175, 1,028, 825, 754, 699 cm^−1^; ^1^H and ^13^C NMR data, see Table 3.

**Table 3:** ^1^H (400 MHz) and ^13^C (100 MHz) NMR data for 9 (Acetone-*d*_6_).

NO.	*δ*_C_, Type	*δ*_H_, mult (*J* Hz)	NO.	*δ*_C_, Type	*δ*_H_, mult (*J* Hz)
1	167.2, C		9	133.4, C	
2	126.9, C		10	130.5, C	
3	131.9, CH	7.88, d (7.2)	11	125.9, C	
4	125.0, CH	7.24, overlapped	12/16	132.0	7.39, d (8.7)
5	133.3, CH	7.51, t (7.2)	13/15	115.4	7.01, d (8.7)
6	121.5, C	7.24, overlapped	14	161.7, C	
7	137.8, C		17	55.8, CH_3_	3.84, s
8	171.5, C		18	35.5, CH_3_	3.13, s

### X-ray single-crystal structure determinations of **2**, **4**, **6**, **7**, and **9**

All of the determined compounds were acquired from the solvent of MeOH by vapor diffusion. The structure elucidation of these compounds was settled by the Sheldrick (2014) software package through direct methods and were refined using least squares minimization. The crystallographic data of compounds **2** (CCDC 2002826), **4** (CCDC 2002827), **6** (CCDC 2002870), **7** (CCDC 2002825), and **9** (CCDC 2002821) have been already deposited in the Cambridge Crystallographic Data Centre.^1^

#### Crystal data for **2**

C_21_H_23_N_3_O_4_, *M*_r_ = 381.42, monoclinic, space group *C*2, *a* = 15.8834 (11) Å, *b* = 6.8528 (7) Å, *c* = 18.4959 (17) Å, *V* = 2013.0 (3) Å^3^, *T* = 293 (2) K, *Z* = 4, *μ*(Cu K*α*) = 0.723 mm^−1^, *D*_calcd_ = 1.259 g/cm^3^. *α* = *γ* = 90°, *β* = 90.565 (8)°. In total, 3,289 reflections were measured (9.56 ≤ 2θ ≤ 132.08), with 2,354 independent unique reflections (*R*_int_ = 0.0466). The final refinement [I ≥ 2*σ* (*I*)] gave *R*_1_ = 0.0633 and *wR*_2_ = 0.1351. The final refinement presented *R*_1_ = 0.1103 and *wR*_2_ = 0.1650 (all data). Flack parameter = 0.0 (9).

#### Crystal data for **4**

C_17_H_15_NO_3_, *M*_r_ = 281.30, monoclinic, space group *C*2/c, *a* = 26.002 (2) Å, *b* = 7.4221 (8) Å, *c* = 14.8450 (13) Å, *V* = 2787.6 (4) Å^3^, *T* = 293 (2) K, *Z* = 8, *μ*(Cu K*α*) = 0.092 mm^−1^, *D*_calcd_ = 1.341 g/cm^3^. *α* = *γ* = 90°, *β* = 103.343 (2)°. In total, 5,145 reflections were measured (5.64 ≤ 2θ ≤50.02), with 2,453 independent unique reflections (*R*_int_ = 0.1063). The final refinement [I ≥ 2*σ* (*I*)] gave *R*_1_ = 0.1063, and *wR*_2_ = 0.1351. The final refinement presented *R*_1_ = 0.0951 and *wR*_2_ = 0.1400 (all data).

#### Crystal data for **6**

C_17_H_15_NO_4_, *M*_r_ = 297.30, monoclinic crystal, space group P2_1_/n, *a* = 8.7113 (5) Ǻ, *b* = 9.5303 (7) Ǻ, *c* = 17.3892 (12) Ǻ, *α* = *γ* = 90°, *β* = 103.465 (2)°, *V* = 1403.99 (16) Ǻ^3^, *T* = 293 (2) K, *Z* = 4, *μ*(Cu K*α*) = 0.834 mm^−1^, *D*_calc_ = 1.407 g/cm^3^. In total, 4,326 reflections were measured (10.46 ≤ 2*θ* ≤ 132.18), with 2,454 independent unique reflections (*R*_int_ = 0.0221). The final refinement [I ≥ 2*σ* (*I*)] gave *R*_1_ = 0.0483, and *wR*_2_ = 0.1334. The final refinement presented *R*_1_ = 0.0608 and *wR*_2_ = 0.1451 (all data).

#### Crystal data for **7**

C_22_H_24_NO_5_, *M*_r_ == 382.42, orthorhombic crystal, space group P2_1_2_1_2_1_, *a* = 9.6170 (4) Ǻ, *b* = 13.3217 (5) Ǻ, *c* = 15.7153 (6) Ǻ, *α* = *β* = *γ* = 90°, *V* = 2013.36 (14) Ǻ^3^, *T* = 293 (2) K, *Z* = 4, *μ*(Cu K*α*) = 0.732 mm^−1^, *D*_calc_ = 1.262 g/cm^3^. In total, 4,449 reflections were measured (8.70 ≤ 2*θ* ≤ 132.78), with 2,991 independent unique reflections (*R*_int_ = 0.0261). The final refinement [I ≥ 2*σ* (*I*)] gave *R*_1_ = 0.0498, and *wR*_2_ = 0.1377. The final refinement gave *R*_1_ = 0.0538, and *wR*_2_ = 0.1422 (all data). Flack parameter = −0.27 (18).

#### Crystal data for **9**

C_18_H_16_N_2_O_3_, *M*_r_ = 308.33, orthorhombic crystal, space group *P*bca, *a* = 15.8992 (14) Ǻ, *b* = 8.5824 (8) Ǻ, *c* = 23.758 (2) Ǻ, *α* = *β* = *γ* = 90°, *V* = 3241.8 (5) Ǻ^3^, *T* = 298 (2) K, *Z* = 8, *μ*(Mo K*α*) = 0.087 mm^−1^, *D*_calc_ = 1.263 g/cm^3^. In total, 14,810 reflections were measured (5.12 ≤ 2*θ* ≤ 50.04), with 2,861 independent unique reflections (*R*_int_ = 0.0842). The final refinement [*I* ≥ 2σ (*I*)] gave *R*_1_ = 0.0516, and *wR*_2_ = 0.1024. The final refinement presented *R*_1_ = 0.1094 and *wR*_2_ = 0.1146 (all data).

### Biological assay

The antimicrobial activity of the isolated compounds was examined by the broth microdilution method according to the method as previously described (Ma et al., 2019). Microorganisms evaluated in this work included *Staphylococcus aureus*, *Escherichia coli*, and *Candida albicans*, and chloramphenicol or ketoconazole was applied as positive control.

## Results and discussion

**Figure 2:**
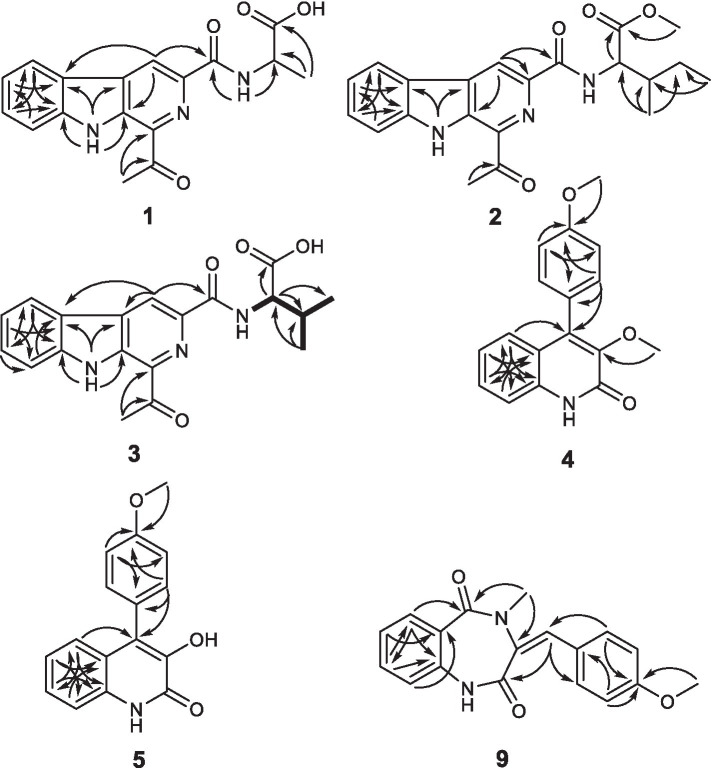
Key HMBC correlations of **1–5** and **9**, and key COSY for **3**.

**Figure 3:**
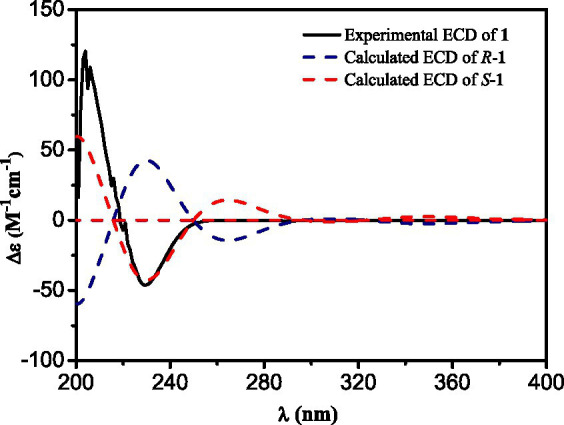
Experimental and calculated ECD spectra of compound **1**.

Compound **1** was obtained as a yellowish needle. The molecular formula of C_17_H_15_N_3_O_4_ was established from the deprotonated-ion HRESIMS at *m/z* 324.0993 (calcd for C_17_H_14_N_3_O_4_, 324.0990). It indicated a *β*-carboline chromophore by the feature of the UV absorptions at 377, 286 and 220 nm (Chen et al., 2010). The ^1^H NMR spectrum in Table 1 showed four aromatic hydrogen signals at *δ*_H_ 8.46 (d, *J* = 7.5 Hz), 7.85 (d, *J* = 8.2 Hz), 7.64 (t, *J* = 7.5 Hz), and 7.35 (t, *J* = 7.5 Hz). The coupling patterns of these four aromatic hydrogens indicated the characteristics of an *ο*-disubstituted benzene ring in the indole moiety (Tangella et al., 2018). The doublet methyl at *δ*_H_ 1.52, multiplet methine at *δ*_H_ 4.59, and the doublet imino proton at *δ*_H_ 8.85 were suggestive of an alanine residue functionality. The ^13^C NMR data showed eleven aromatic carbon signals from *δ*_C_ 142.4 to 113.3 (Table 1), which featured the *β*-carboline skeleton. The signals of keto carbonyl at *δ*_C_ 200.9 and the methyl at *δ*_C/H_ 25.9/2.93, along with the HMBC crosspeaks from the methyl to the carbonyl, implied the existence of an acetyl group (Figure 2). It was located at C-1 on the basis of correlation from H-15 to C-1. Further interactions from H-17 to C-16 and C-19, and from H-4 to C-16 suggested the linkage of C-3 and the alanine residue through C-16. Thus, the plain structure of **1** was accomplished. Considering that alanine moiety plays a crucial role in the specific optical rotation in compound **1** (the same condition also applied to compounds **2**, **3,** and **10**), the consistent specific rotation behaviors of **1** {[ $\alpha {]}_{D}^{20}\;$ + 10.8 (*c* 0.062, MeOH)}and oldhamiaine A {[ $\alpha {]}_{D}^{25}$ + 14.0 (*c* 0.062, MeOH)}suggested the absolute configuration of C-19 to be *S* (Zhang et al., 2015). Additionally, the experimental and calculated ECD curves (Figure 3) further supported the absolute configuration assignment. The absolute stereochemistry of **1** was established to be 19*S*.

**Figure 4:**
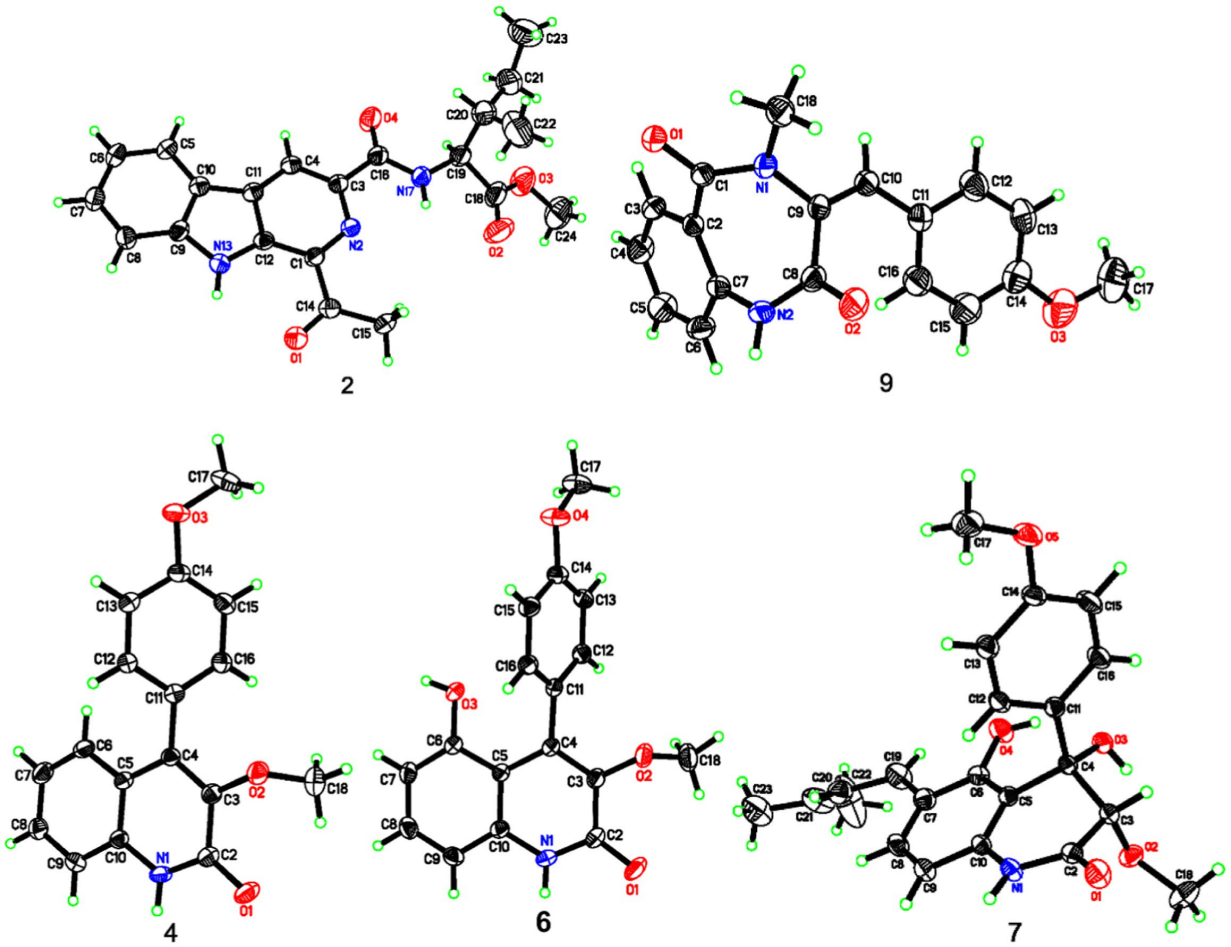
X-ray ORTEP diagrams of **2**, **4**, **6**, **7**, and **9**.

Compound **2** was purified as light yellow needles with a molecular formula of C_21_H_23_N_3_O_4_ given by the positive HRESIMS at *m/z* 382.1762 [M + H]^+^ (calcd for C_21_H_24_N_3_O_4_, 382.1761). The UV and IR spectra of **2** also showed the absorption features of a *β*-carboline skeleton. The ^1^H and ^13^C NMR spectroscopic data of **2** (Table 1) in the downfield regions were almost identical with those of **1**, indicative of the same *β*-carboline moiety. Furthermore, the signals in the upfield of two methines at *δ*_H_ 4.60 and 2.10, one diastereotopic methylene at *δ*_H_ 1.54 and 1.31, two methyls at *δ*_H_ 0.98 and 0.95, and the carbon resonances at *δ*_C_ 56.8, 37.2, 25.4, 16.6, 11.7 (Table 1), suggested the fragment of an isoleucine residue, which was supported by the HMBC correlations (Figure 2). Finally, the X-ray analyses unambiguously determined the absolute configuration of **2** (Figure 4).

Compound **3**, light yellow needles, was determined to have a molecular formula of C_19_H_19_N_3_O_4_ by the negative HRESIMS data (*m/z* 352.1302, calcd for C_19_H_18_N_3_O_4_, 352.1303). Its UV and IR absorption bands were almost the same as those of compounds **1** and **2**. The spectroscopic data covering ^1^H and ^13^C NMR (Table 1) closely resembled those of **1**, except for additional appearances of one methine and one methyl signals. Careful analyses of the chemical shifts and the splitting behaviors of a double doublets methine at *δ*_H_ 4.52, a multiplet methine at *δ*_H_ 2.34, two doublet methyl groups at *δ*_H_ 1.03, and a doublet NH at *δ*_H_ 8.59 were suggestive of a valine motif, which was supported by the corresponding ^1^H-^1^H COSY data (Figure 2). The position of the valine motif was accomplished according to the HMBC interactions from H-4 and H-17 to C-16 (Figure 2). The acetyl group linked at C-1 was elucidated by the correlations from H-15 to C-1 and C-14. The similar ECD absorptions (ESI Supplementary Figure S24) together with evidence of the same optical rotation direction with that of **10** confirmed the absolute configuration of **3.**

Compound **4**, colorless needles, exhibited a deprotonated ion signal at *m*/*z* 280.0973 (calcd for C_17_H_14_NO_3_, 280.0968) in negative HRESIMS, which corresponded with its molecular formula of C_17_H_15_NO_3_. In ^1^H NMR spectroscopic data, two aromatic protons at *δ*_H_ 7.44 and 7.18 (Table 2) which were presented in an *ortho*-coupled doublet fashion, indicated the presence of a *p*-disubstituted ring fragment. Four aromatic protons at *δ*_H_ 7.60, 7.48, 7.39, and 7.15 (Table 2) represented the existence of an *o*-disubstituted phenyl ring moiety. Evidence of the HMBC data from H-17 to C-14 and from H-12 to C-4 and C-14 revealed the methoxyl and C-4 anchored at the *p*-disubstituted phenyl ring (Figure 2). The NH group and C-4 were located at the *o*-disubstituted phenyl ring, which was supported by HMBC cross peaks from H-6 to C-4 and C-10, along with the chemical shift of C-10. The remaining three carbon signals, including a carbonyl at *δ*_C_ 160.3, a quaternary carbon at *δ*_C_ 147.0, and a methoxyl at *δ*_C_ 60.3 (Table 2) were assembled to complete the whole structure. Finally, the proposed structure of **3** was further undoubtedly defined by the X-ray diffraction analysis (Figure 4).

The structure of **5** was finally determined by 1D and 2D NMR data (Figure 2) along with the data from a previously reported synthetic intermediate (He et al., 2015; Zhang et al., 2015), and it was purified from natural for the first time.

Compound **9** was named dehydrocyclopeptin (Hayashi et al., 1997; Ma et al., 2012). It was first isolated from *Penicillium cyclopium* (Framm et al., 1973), but the chemical shift assignments of some signals and the configuration of its double band needed to be discussed. In the current report, HSQC and HMBC spectra (Figure 2) helped to provide the ^1^H and ^13^C NMR data (Table 3), and X-ray diffraction analysis unambiguously built up an *E* configuration of the double band (Figure 4).

The rest of the compounds, namely, **6**, **7**, **8,** and **10**, were determined to be pesimquinolone R; peniprequinolone; 4,5-dihydroxy-3,4-dihydro-3-methoxy-4-(4′-methoxyphenyl)-2(1*H*)-quinolinone; and tunicoidine F, respectively, through comparison of the corresponding spectroscopic data with the literature. Among them, we first reported the X-ray data for compounds **6**, **7,** and **9**.

The antimicrobial activity of compounds **1**–**10** was examined against *S. aureus*, *E. coli,* and *C. albicans* with chloramphenicol or ketoconazole as the positive control. As the results show in Table 4, compound **1** demonstrated an obvious antimicrobial effect, while **3** and **4** exhibited activity toward *S. aureus* and *E. coli*.

**Table 4:** Antimicrobial activity of compounds 1–10 (MIC *μ*g/mL).

	1	2	3	4	5	6	7	8	9	10	Control
*S. aureus*	8.0	>200	50.0	12.5	>200	>200	>200	>200	>200	>200	3.50*^a^*
*E. coli*	5.0	>200	25.0	50.0	–	–	–	–	–	–	7.50*^a^*
*C. albicans*	2.0	–	>200	>200	–	–	–	>200	>200	>200	7.50*^b^*

^a^Chloramphenicol. ^b^Ketoconazole. –, no activity.

## Conclusion

In summary, an OSMAC-based strategy applied to *P. raistrickii* of changing cultivation status from shaking fermentation to a static condition in a liquid medium expanded the alkaloidal SM diversity and thus induced the accumulation of four unreported alkaloids, including three *β*-carbolines (**1**–**3**) and one 2-quinolinone (**4**), along with one new natural compound 2-quinolinone (**5**) and five known alkaloids (**6**–**10**). The outcome indicated a big effect from a small change in the OSMAC strategy application, showing it to be a powerful tool in unlocking the fungal cryptic BGCs to obtain novel natural products.

*β*-Carboline alkaloids possess the fundamental framework of tricyclic pyrido [3,4-b] indole and are mainly obtained from plants and marine organisms, with only a few from microbes to our knowledge. 2-quinolinones, or named 2-quinolones, are quinoline derivatives with a carbonyl group inserted in the 2 position, most of which are produced by fungal genera (Neff et al., 2012). Benzodiazepine alkaloids are seven-membered cyclodipeptides composed of an anthranilic acid and another *α*-amino acid or its deoxygenated analogs. They are metabolites of filamentous fungi or actinomycetes, with one exception from sea hare up to now, and have not been found in higher plants (Roos, 1990; Ojika et al., 1993). These three kinds of alkaloids have a strong attraction to scientists due to their structural diversities and extensive scope of pharmacological effects, such as antitumor, antidepressant, antimicrobial, antimalarial, anti-inflammatory, antioxidant, insecticidal and analgesic effects (Roos, 1990; Cao et al., 2007; Cui et al., 2009; Wang et al., 2014; Simonetti et al., 2016; Reddy et al., 2018).

The structures of **1**–**10** were constructed by comprehensive analyses of the spectroscopic data. Measures including X-ray diffraction data, ECD, and optical rotation values helped to solve the absolute configurations of the new compounds. In addition, the configuration of the double band in compound **9** was unambiguously revised by X-ray diffraction data, and its ^1^H and ^13^C NMR data were reassigned in this work. Furthermore, it was the first report of the X-ray diffraction analyses on the absolute stereochemistry of compounds **7**.

All of the compounds (**1**–**10**) in the work were screened for their antimicrobial activity against microorganisms like *S. aureus*, *E. coli,* and *C. albicans*. Compounds **1**, **3,** and **4** showed antimicrobial effects. As for the *β*-carbolines obtained in the work, compound **1** exhibited an obvious pharmaceutical effect against tested microorganisms with MIC values of 8.0, 5.0, and 2.0 *μ*g/mL, and compound **3** also showed some activity. The results implied that the amino acid residues and a free carboxyl group in this type of *β*-carboline alkaloids might play a pivotal part in the antimicrobial effect.

## Data availability statement

The datasets generated for this study can be found in the online repositories. The names of the repository/repositories and accession number(s) can be found in the article/supplementary material.

## Author contributions

MZ: Data curation, Investigation, Writing – original draft. HK: Investigation, Writing – original draft, Formal analysis. WL: Writing – original draft, Resources, Supervision, Validation, Visualization, Writing – review & editing. LM: Investigation, Writing – original draft, Formal analysis, Methodology. DL: Funding acquisition, Project administration, Supervision, Validation, Writing – review & editing.

## Footnotes

^1^www.ccdc.cam.ac.uk/data_request/cif
