# Supplementary material for: Alkaloid diversity expansion of a talent fungus Penicillium raistrichii through OSMAC-based cultivation
Source: Front Microbiol. 2023 Nov 9;14:1279140. doi: 10.3389/fmicb.2023.1279140 (PMC10665910; doi:10.3389/fmicb.2023.1279140)
Supplement: Supplementary file 1 [file Data_Sheet_1.docx]

Supplementary Material

Alkaloids diversity expansion of a talent fungus *Penicillium raistrichii* through OSMAC based cultivation

# List of Supporting Information

[Figure S1. ^1^H NMR spectrum (400 MHz) of **1** in DMSO-*d*_6_. 4](#_Toc147592506)

[Figure S2. ^13^C NMR spectrum (100 MHz) of **1** in DMSO-*d*_6_. 4](#_Toc147592507)

[Figure S3. HSQC spectrum of **1** in DMSO-*d*_6_. 5](#_Toc147592508)

[Figure S4. HMBC spectrum of **1** in DMSO-*d*_6_. 5](#_Toc147592509)

[Figure S5. IR spectrum of **1**. 6](#_Toc147592510)

[Figure S6. UV spectrum of **1** in MeOH. 6](#_Toc147592511)

[Figure S7. HRESIMS of **1**. 7](#_Toc147592512)

[Figure S8. ^1^H NMR spectrum (600 MHz) of **2** in DMSO-*d*_6_. 7](#_Toc147592513)

[Figure S9. ^13^C NMR spectrum (150 MHz) of **2** in DMSO-*d*_6_. 8](#_Toc147592514)

[Figure S10. HSQC spectrum of **2** in DMSO-*d*_6_. 8](#_Toc147592515)

[Figure S11. HMBC spectrum of **2** in DMSO-*d*_6_. 9](#_Toc147592516)

[Figure S12. IR spectrum of **2**. 9](#_Toc147592517)

[Figure S13. UV spectrum of **2** in MeOH. 10](#_Toc147592518)

[Figure S14. HRESIMS of **2**. 10](#_Toc147592519)

[Figure S15. ^1^H NMR spectrum (400 MHz) of **3** in DMSO-*d*_6_. 11](#_Toc147592520)

[Figure S16. ^13^C NMR spectrum (100 MHz) of **3** in DMSO-*d*_6_. 11](#_Toc147592521)

[Figure S17. ^1^H-^1^H COSY spectrum of **3** in DMSO-*d*_6_. 12](#_Toc147592522)

[Figure S18. HSQC spectrum of **3** in DMSO-*d*_6_. 12](#_Toc147592523)

[Figure S19. HMBC spectrum of **3** in DMSO-*d*_6_. 13](#_Toc147592524)

[Figure S20. IR spectrum of **3**. 13](#_Toc147592525)

[Figure S21. UV spectrum of **3** in MeOH. 14](#_Toc147592526)

[Figure S22. HRESIMS of **3**. 14](#_Toc147592527)

[Figure S23. Experimental ECD spectra of **2**, **3** and **7**. 15](#_Toc147592528)

[Figure S24. ^1^H NMR spectrum (500 MHz) of **4** in Py-*d*_5_. 15](#_Toc147592529)

[Figure S25. ^13^C NMR spectrum (125 MHz) of **4** in Py-*d*_5_. 16](#_Toc147592530)

[Figure S26. HSQC spectrum of **4** in Py-*d*_5_. 16](#_Toc147592531)

[Figure S27. HMBC spectrum of **4** in Py-*d*_5_. 17](#_Toc147592532)

[Figure S28. IR spectrum of **4**. 17](#_Toc147592533)

[Figure S29. UV spectrum of **4** in MeOH. 18](#_Toc147592534)

[Figure S30. HRESIMS of **4**. 18](#_Toc147592535)

[Figure S31. ^1^H NMR spectrum (400 MHz) of **5** in DMSO-*d*_6_. 19](#_Toc147592536)

[Figure S32. ^13^C NMR spectrum (100 MHz) of **5** in DMSO-*d*_6_. 19](#_Toc147592537)

[Figure S33. HSQC spectrum of **5** in DMSO-*d*_6_. 20](#_Toc147592538)

[Figure S34. HMBC spectrum of **5** in DMSO-*d*_6_. 20](#_Toc147592539)

[Figure S35. IR spectrum of **5**. 21](#_Toc147592540)

[Figure S36. UV spectrum of **5** in MeOH. 21](#_Toc147592541)

[Figure S37. HRESIMS of **5**. 22](#_Toc147592542)

[Attachment S1. Supporting information for the calculated ECD spectra of compounds **1**. 23](#_Toc147592543)


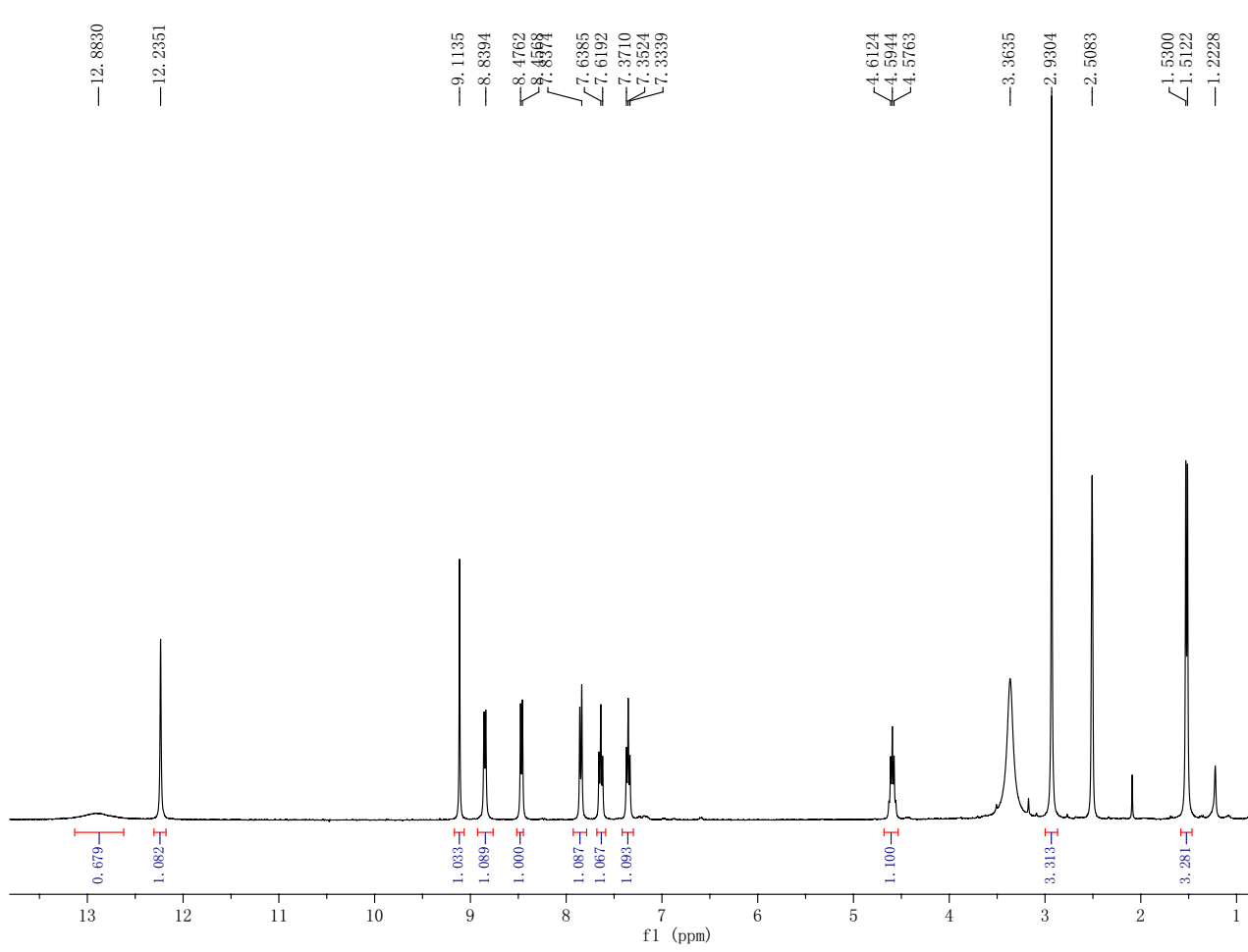


## Figure S1. ^1^H NMR spectrum (400 MHz) of 1 in DMSO-*d*_6_.
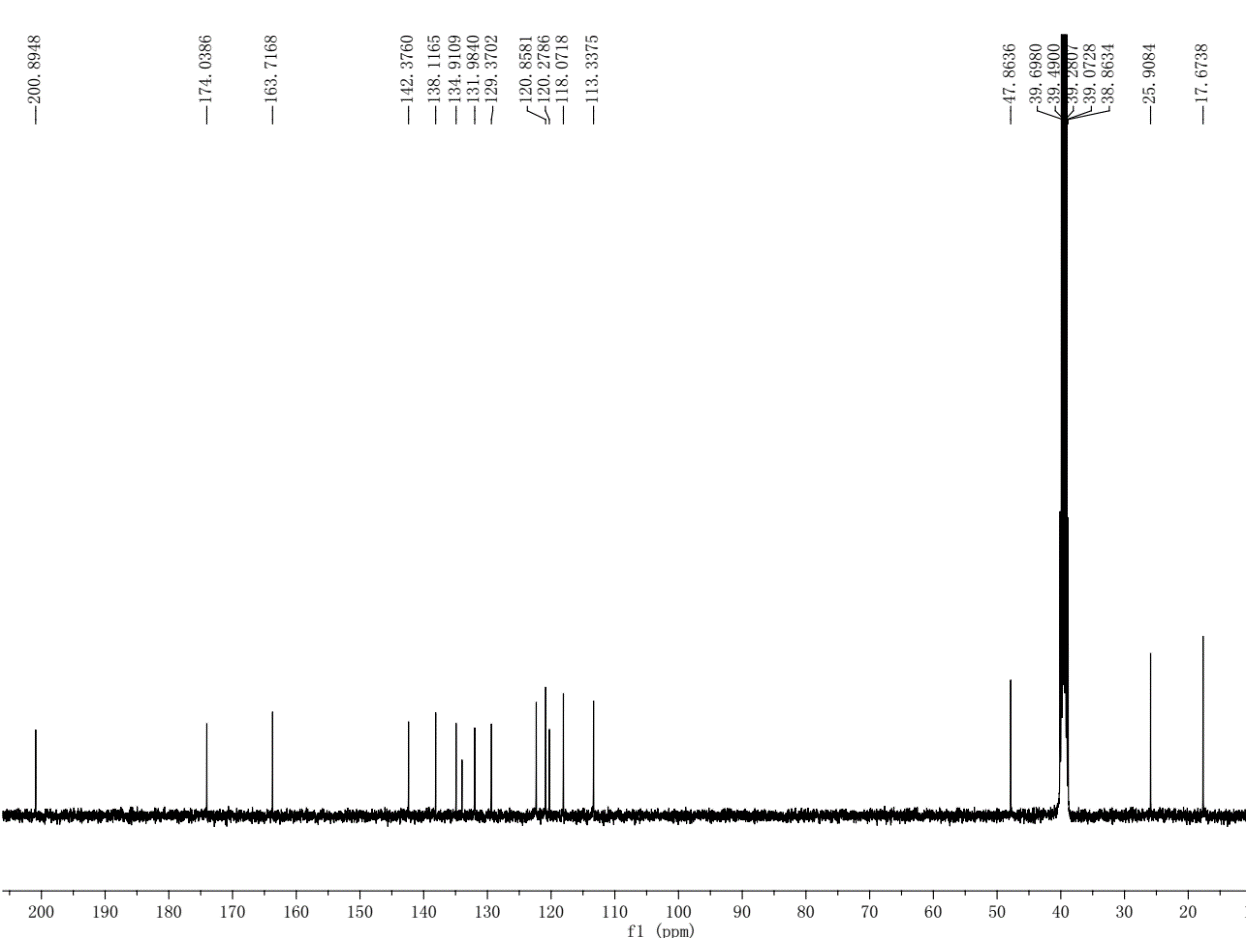


## Figure S2. ^13^C NMR spectrum (100 MHz) of 1 in DMSO-*d*_6_.


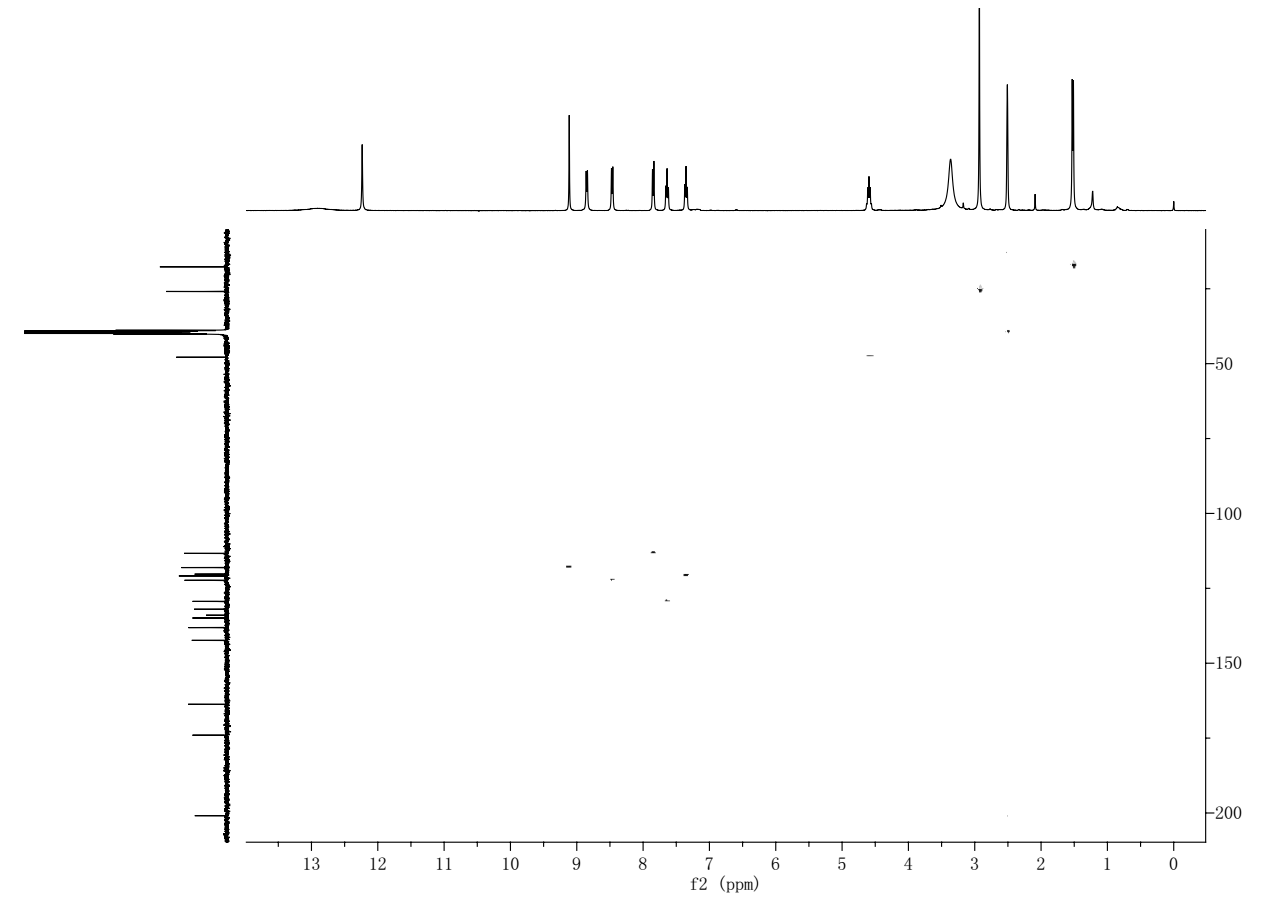


## Figure S3. HSQC spectrum of 1 in DMSO-*d*_6_.


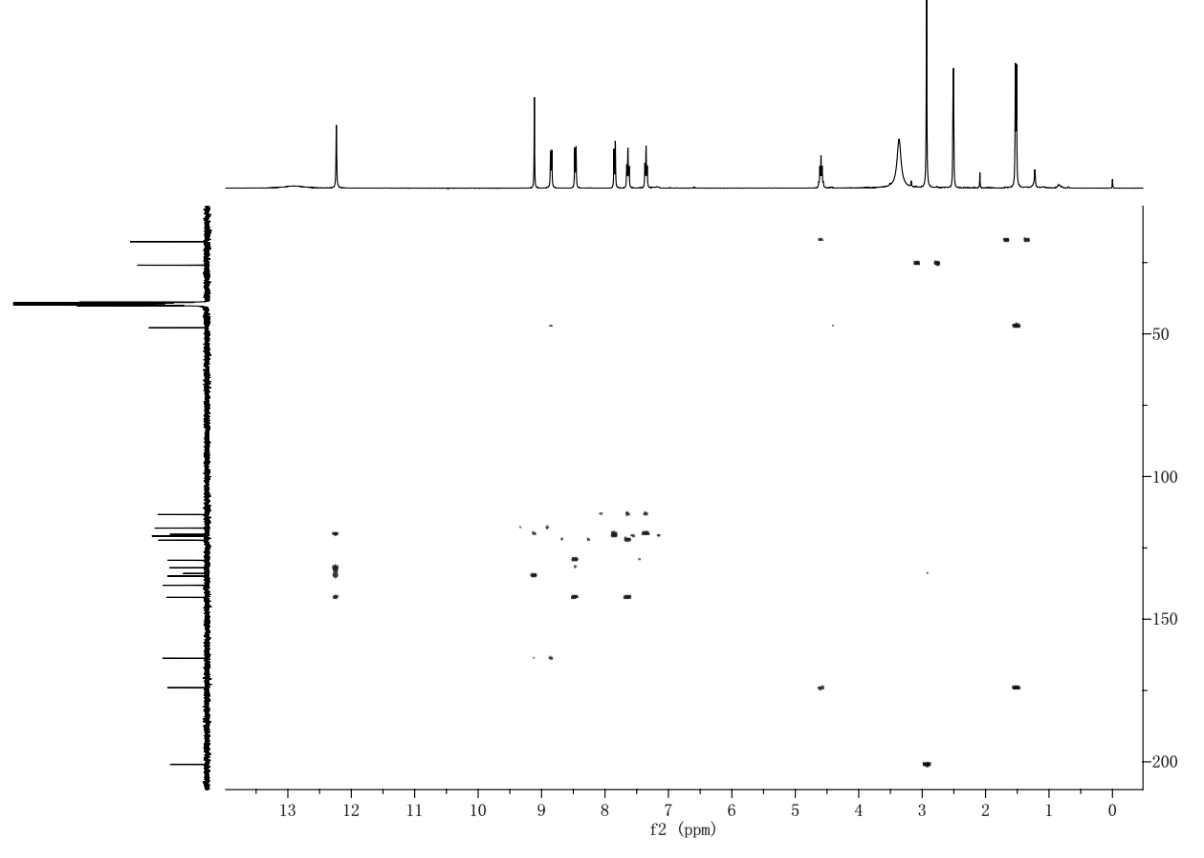


## Figure S4. HMBC spectrum of 1 in DMSO-*d*_6_.


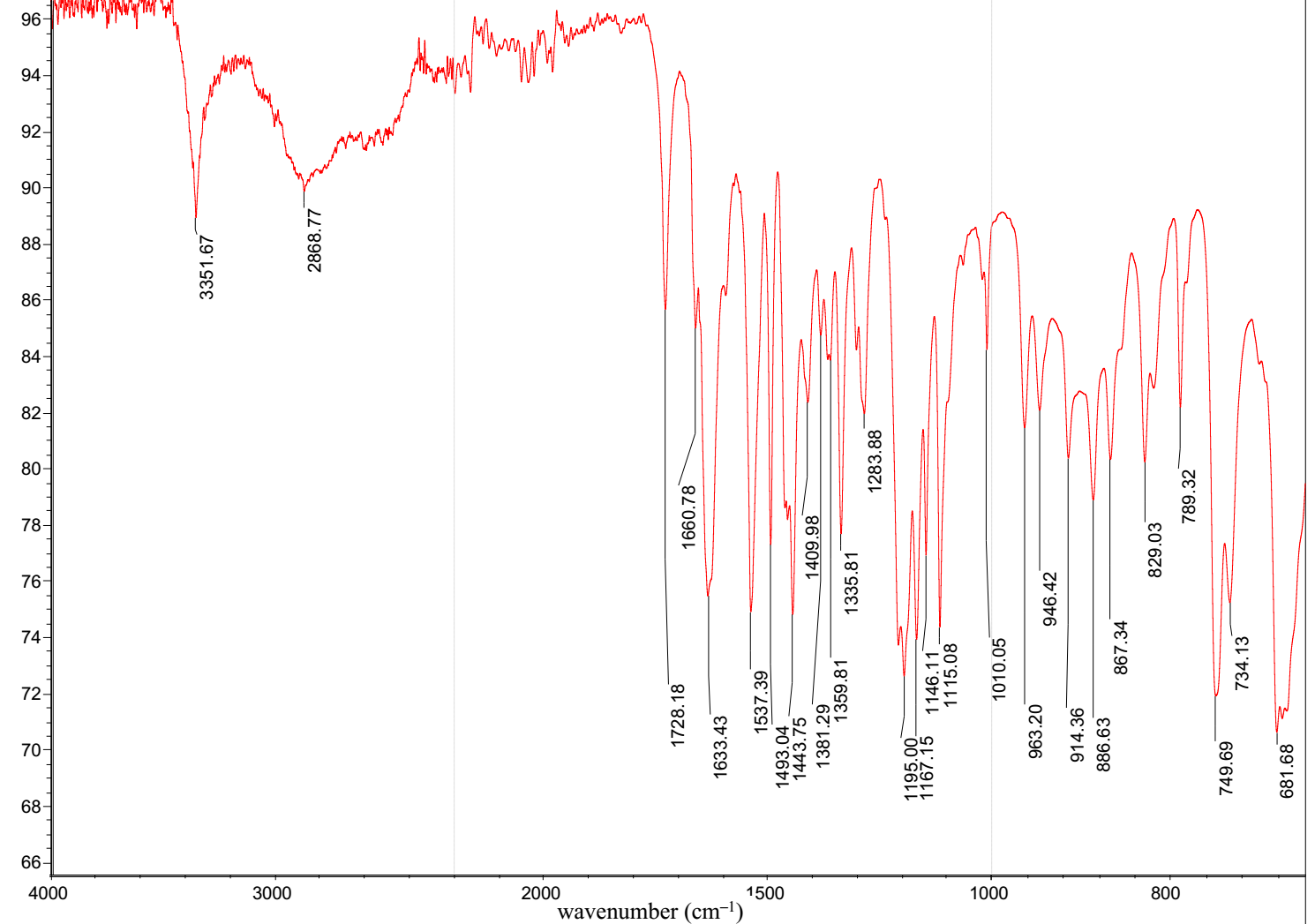


## Figure S5. IR spectrum of 1.


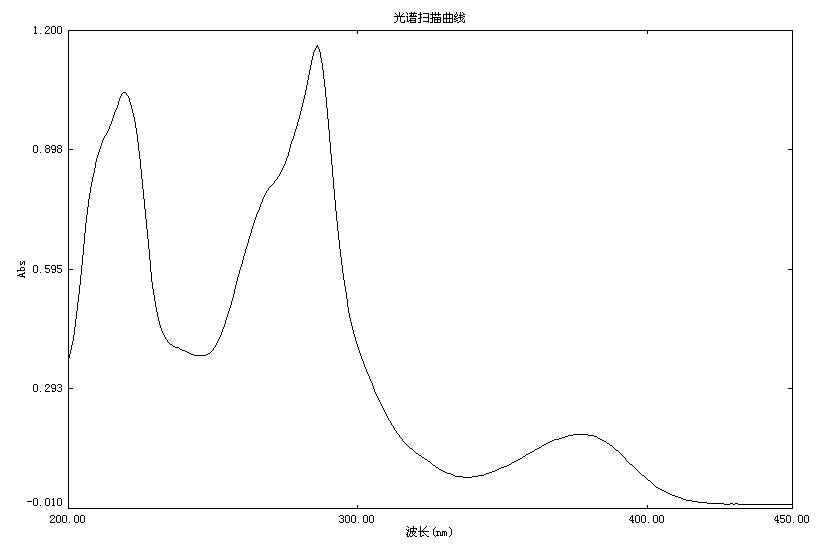


wavelength (nm)

## Figure S6. UV spectrum of 1 in MeOH.


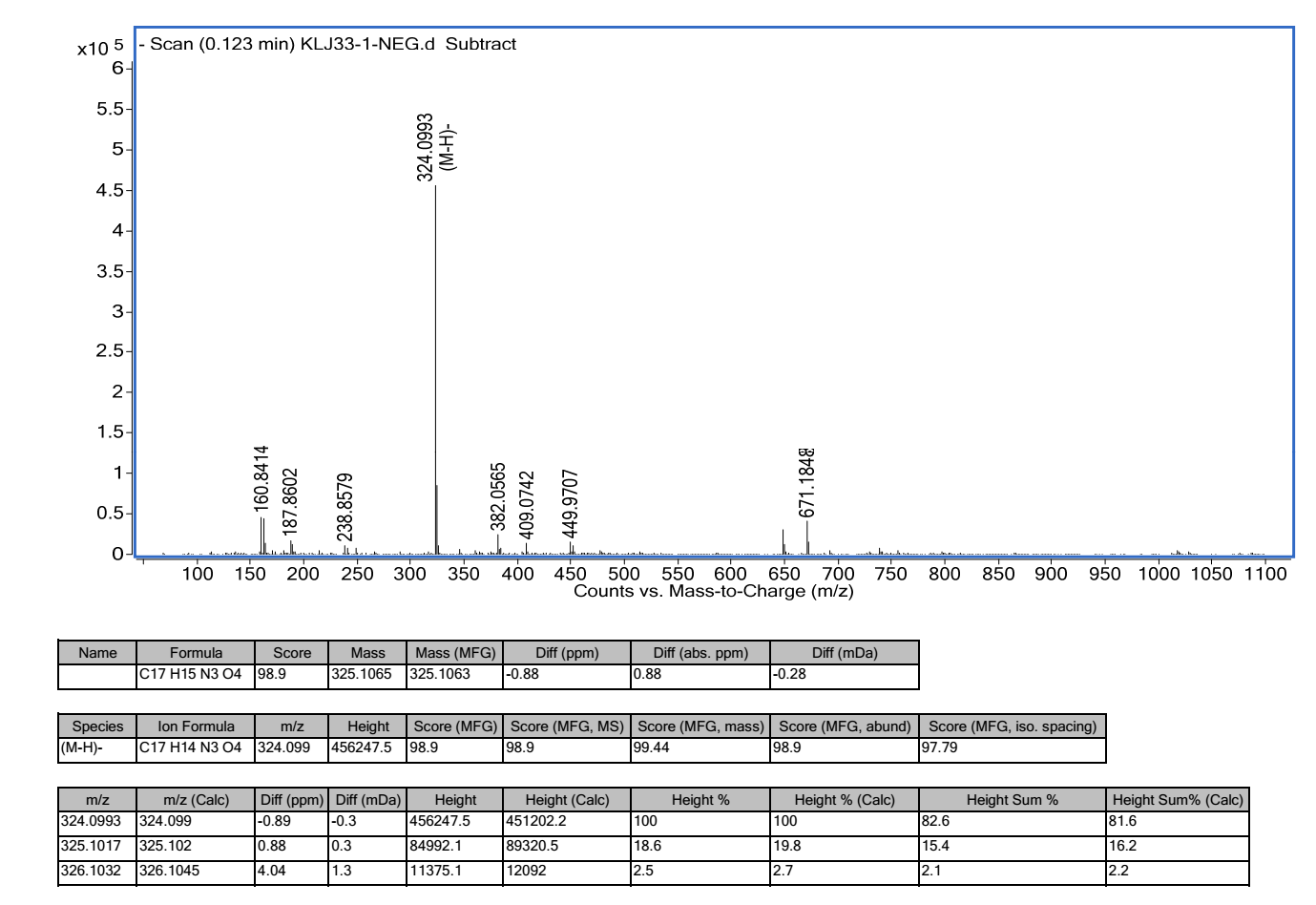


## Figure S7. HRESIMS of 1.


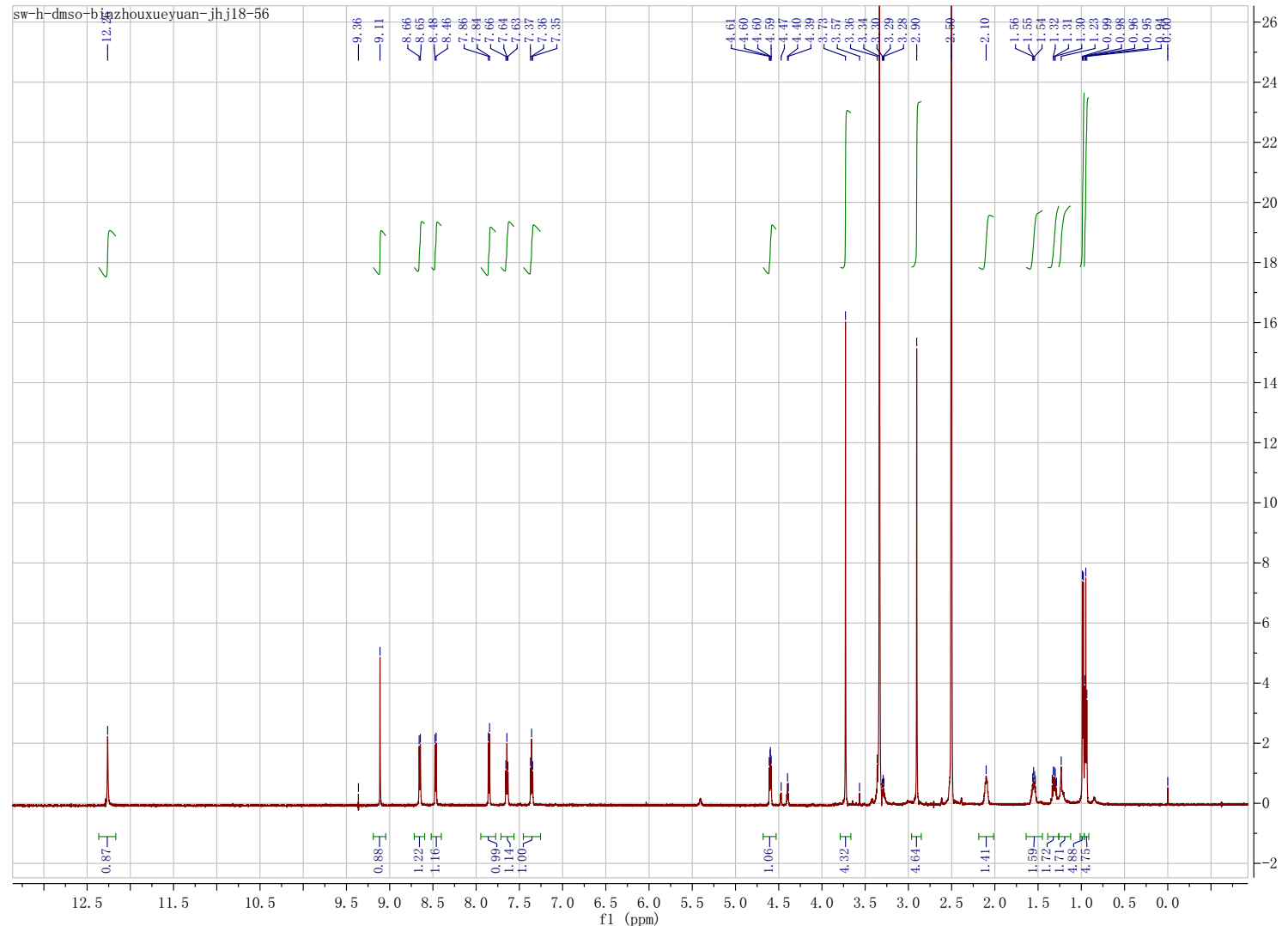


## Figure S8. ^1^H NMR spectrum (600 MHz) of 2 in DMSO-*d*_6_.


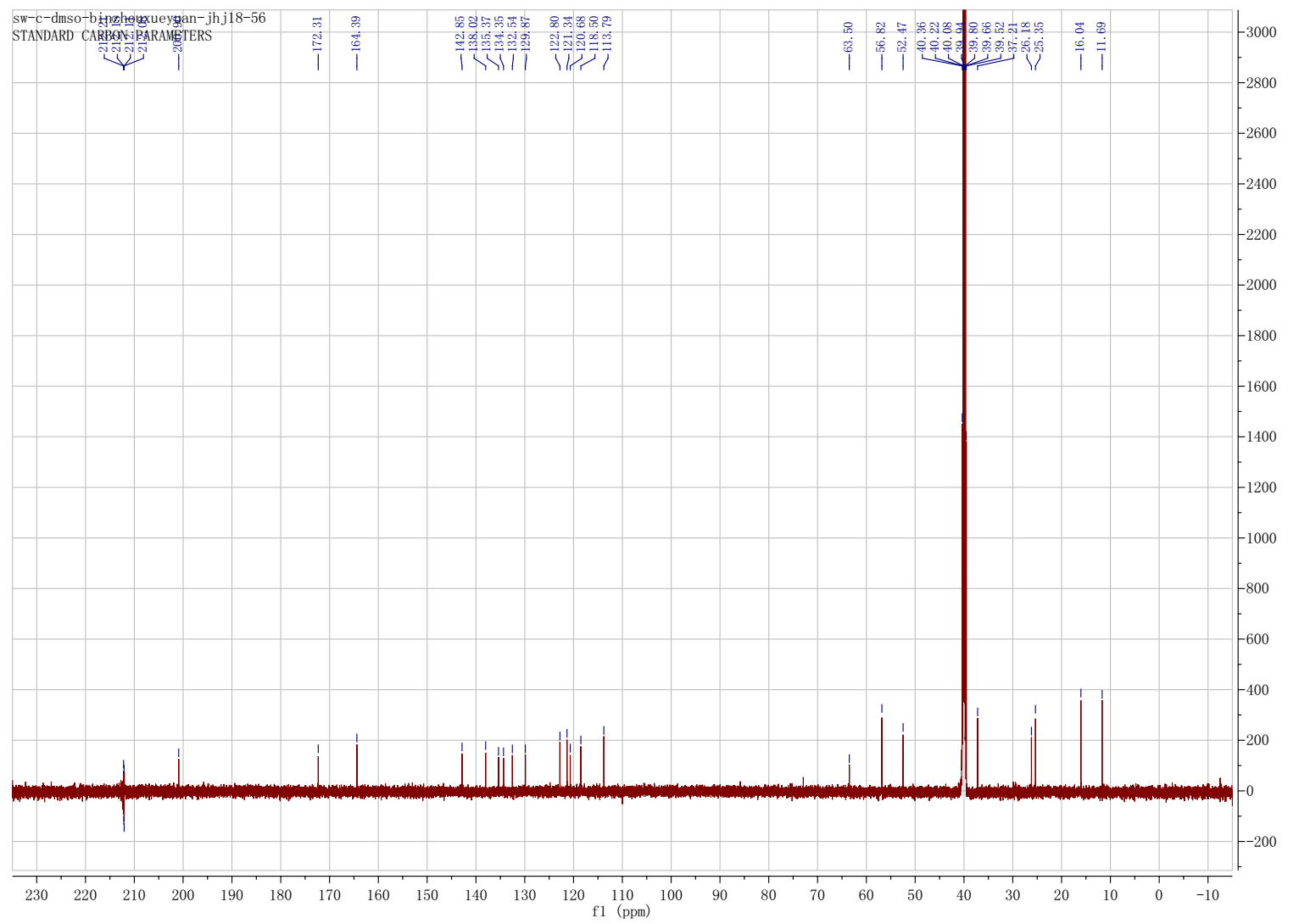


## Figure S9. ^13^C NMR spectrum (150 MHz) of 2 in DMSO-*d*_6_.


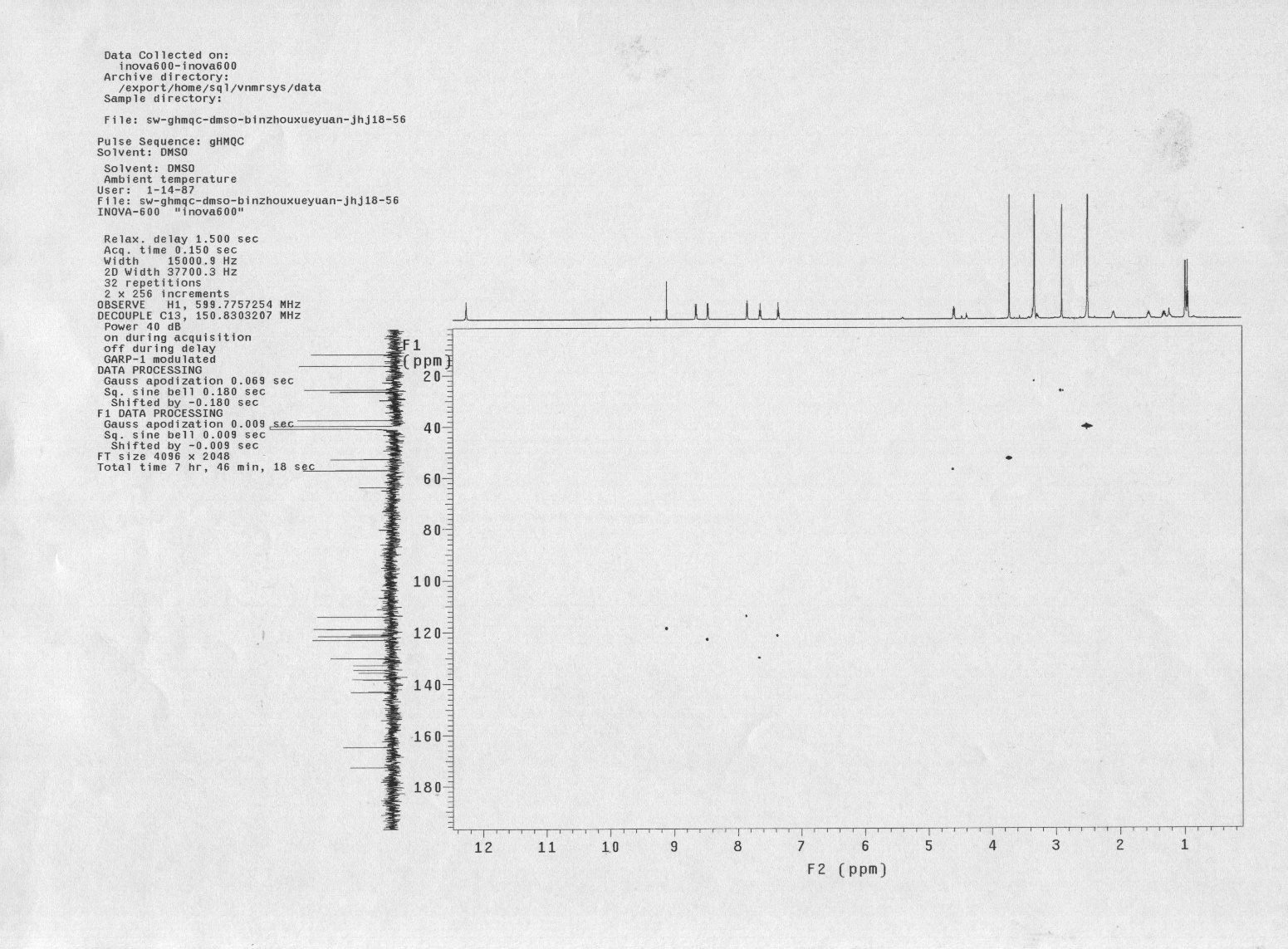


## Figure S10. HSQC spectrum of 2 in DMSO-*d*_6_.


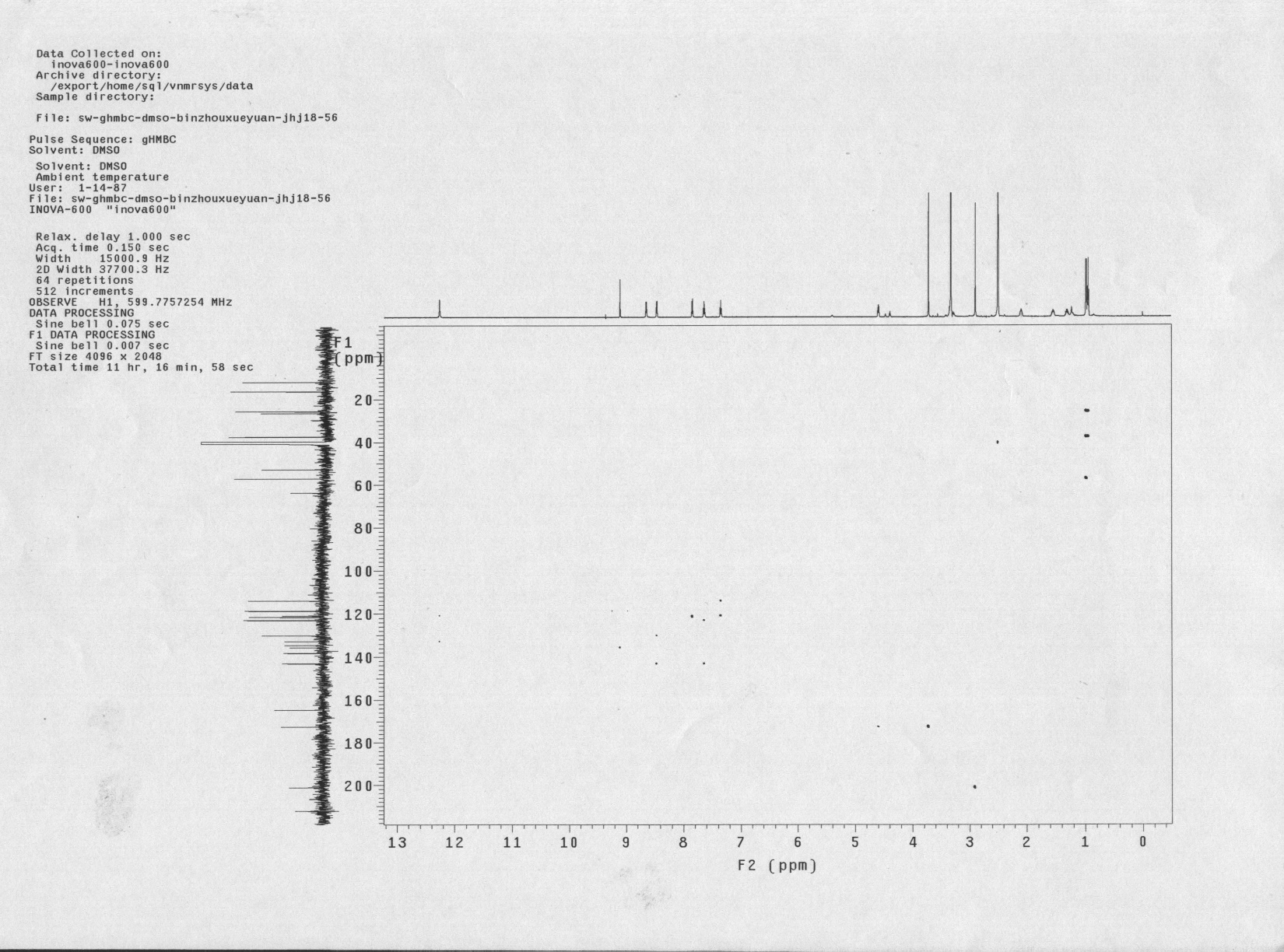


## Figure S11. HMBC spectrum of 2 in DMSO-*d*_6_.


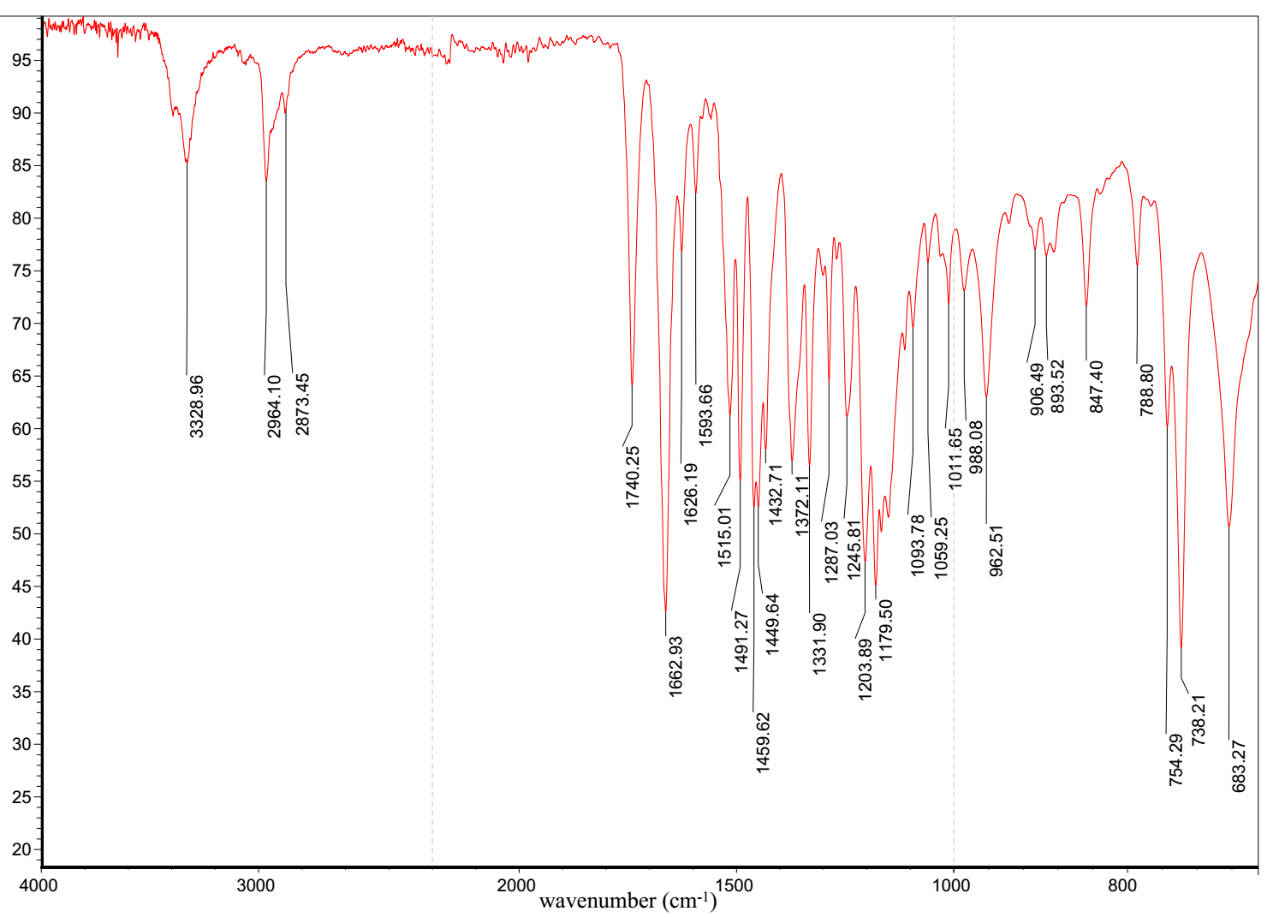


## Figure S12. IR spectrum of 2.


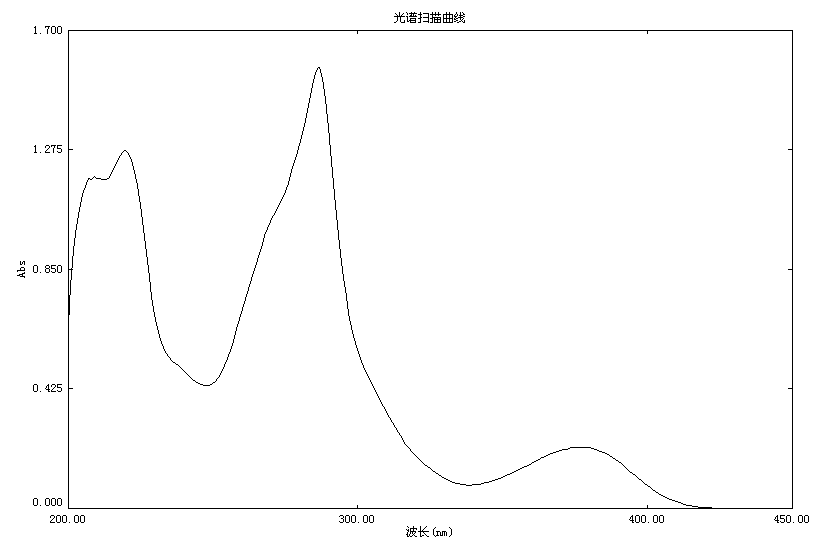


wavelength (nm)

## Figure S13. UV spectrum of 2 in MeOH.


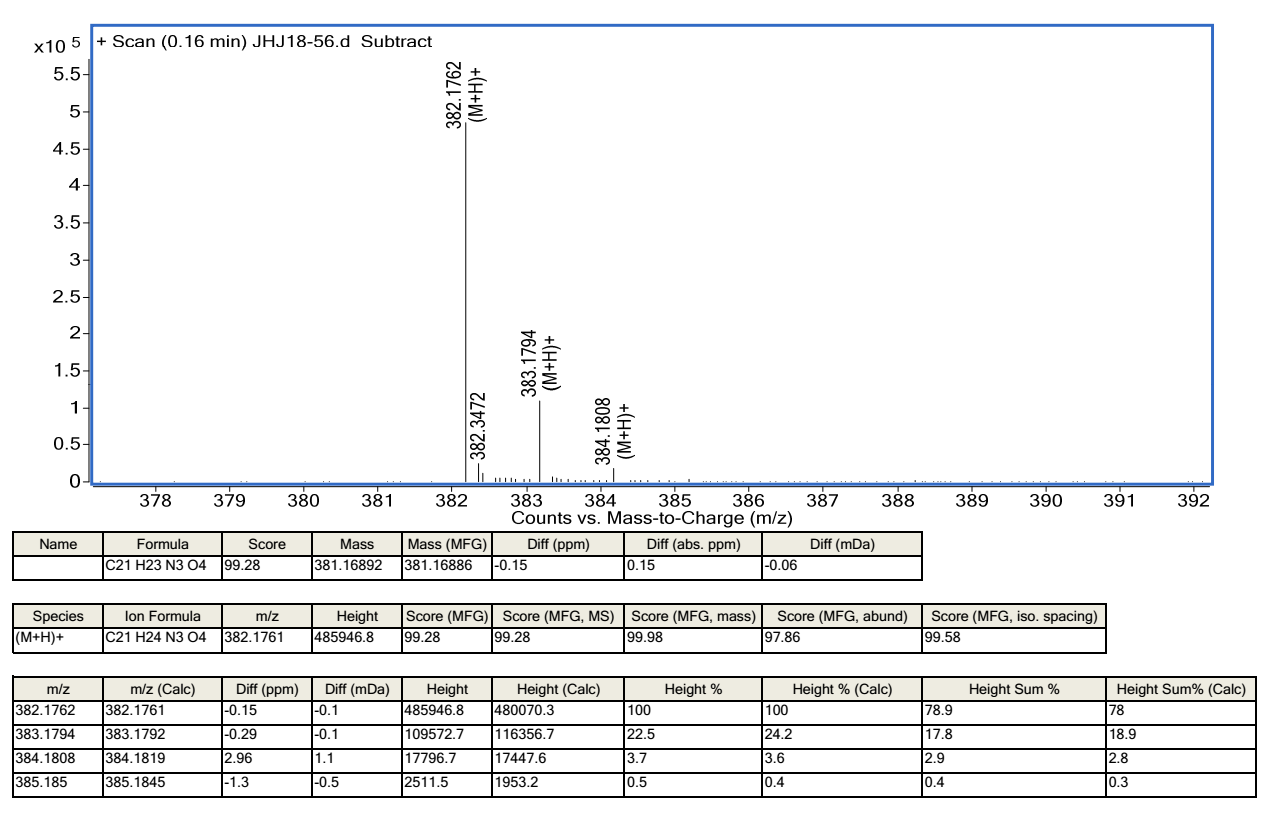


## Figure S14. HRESIMS of 2.


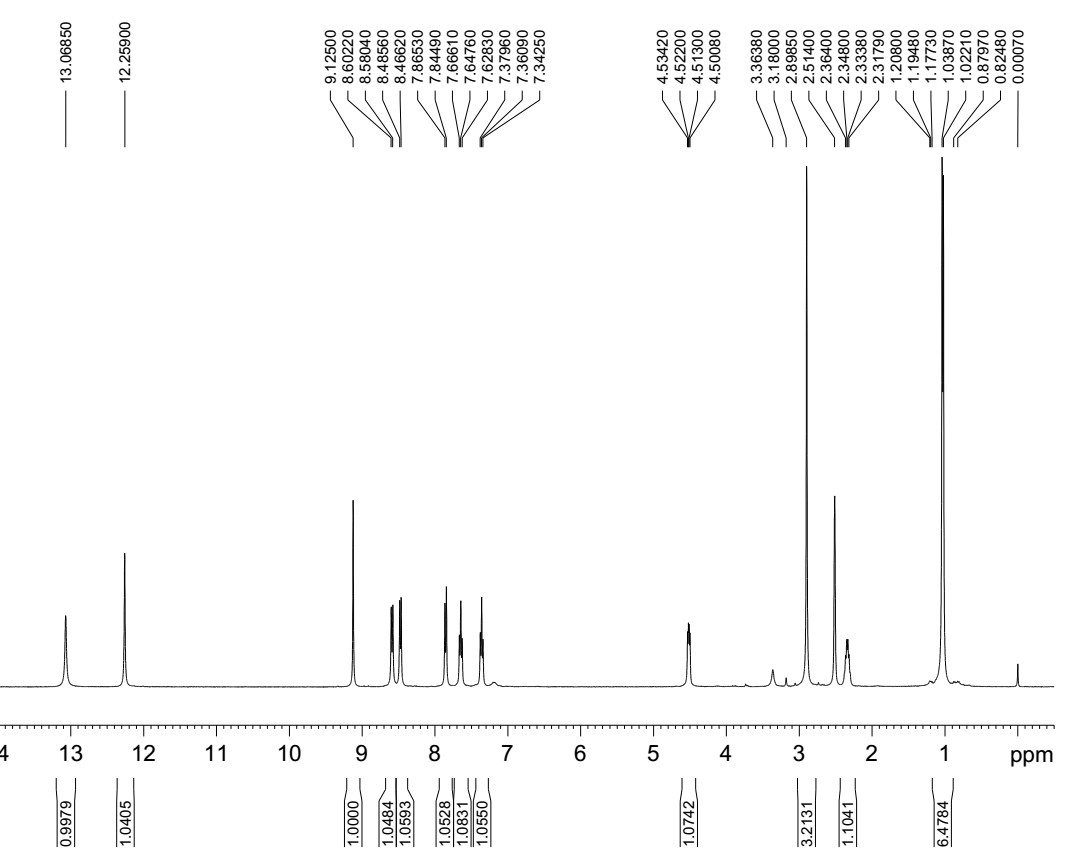


## Figure S15. ^1^H NMR spectrum (400 MHz) of 3 in DMSO-*d*_6_.


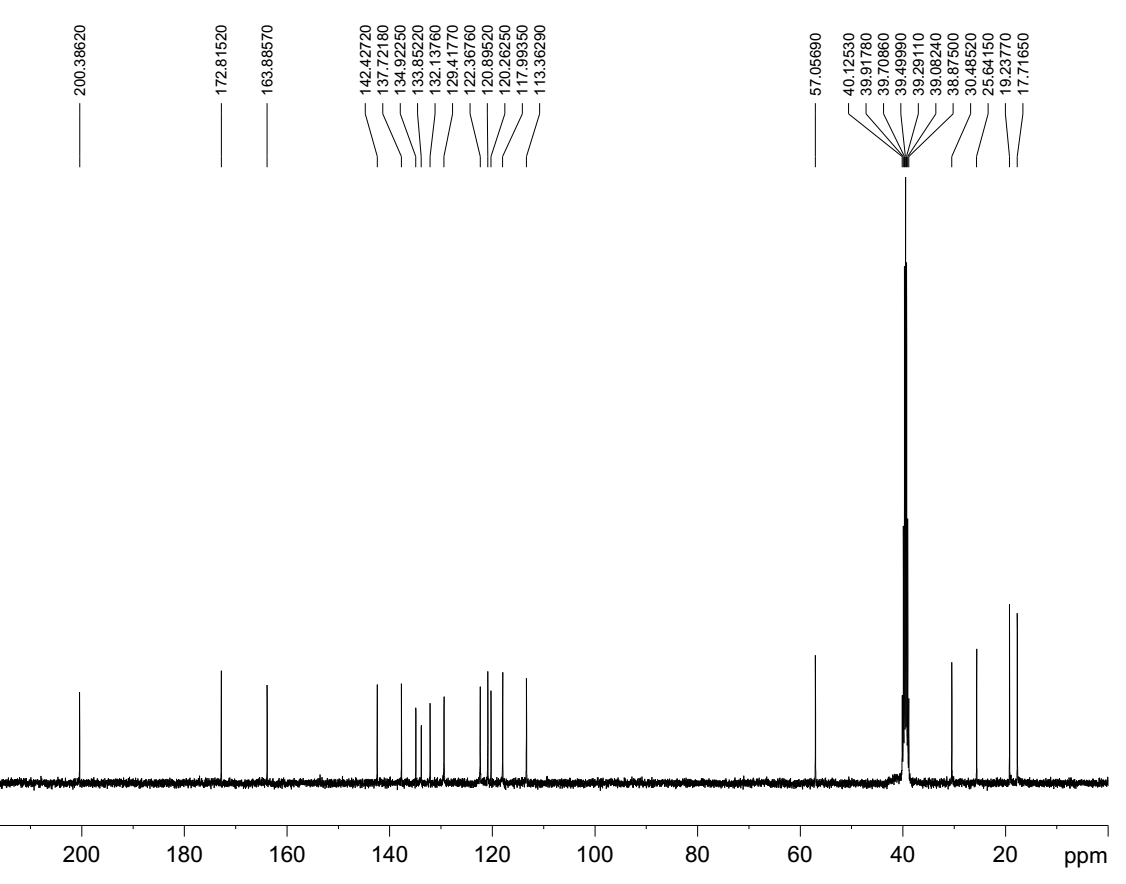


## Figure S16. ^13^C NMR spectrum (100 MHz) of 3 in DMSO-*d*_6_.


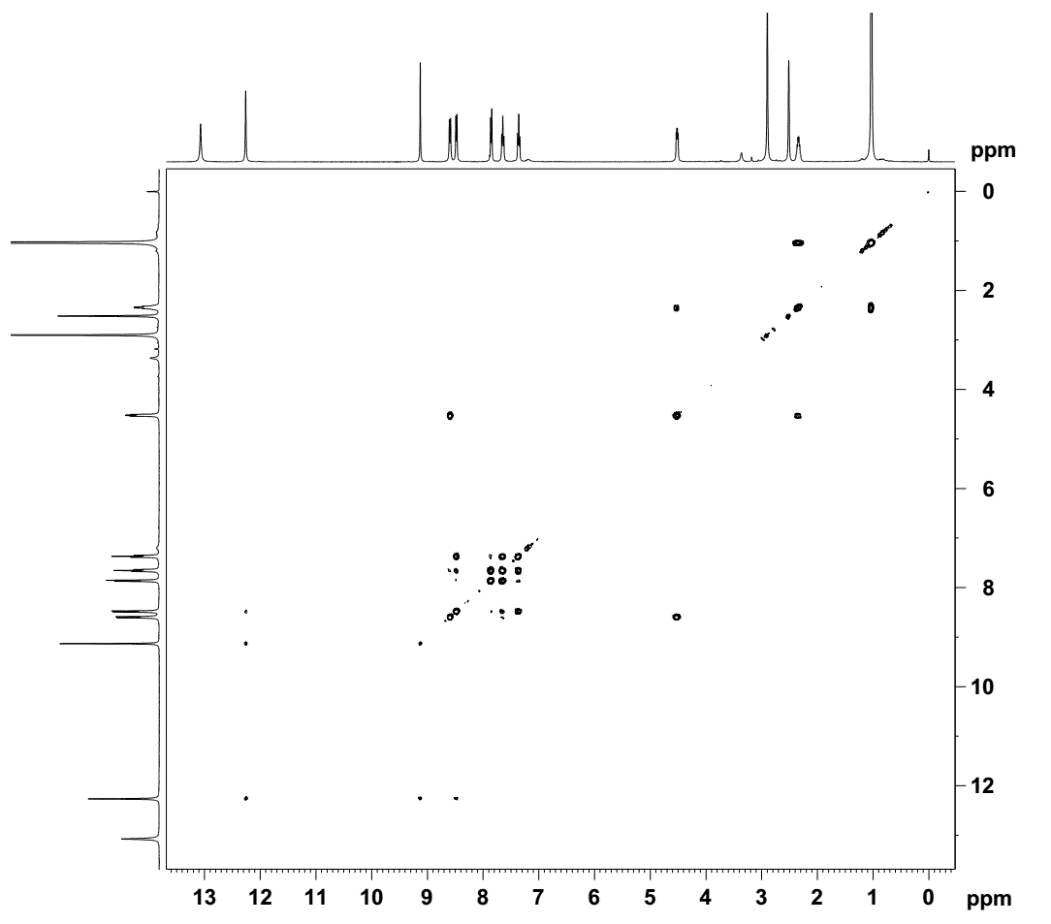


## Figure S17. ^1^H-^1^H COSY spectrum of 3 in DMSO-*d*_6_.


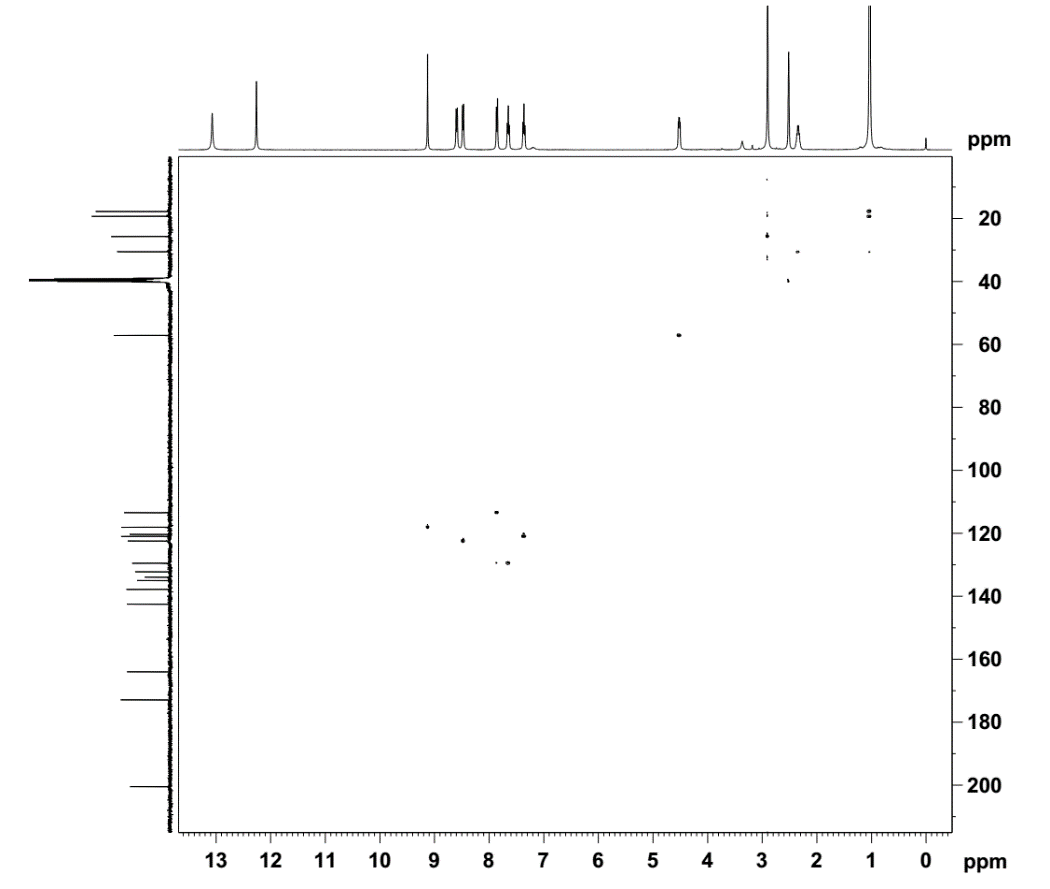


## Figure S18. HSQC spectrum of 3 in DMSO-*d*_6_.


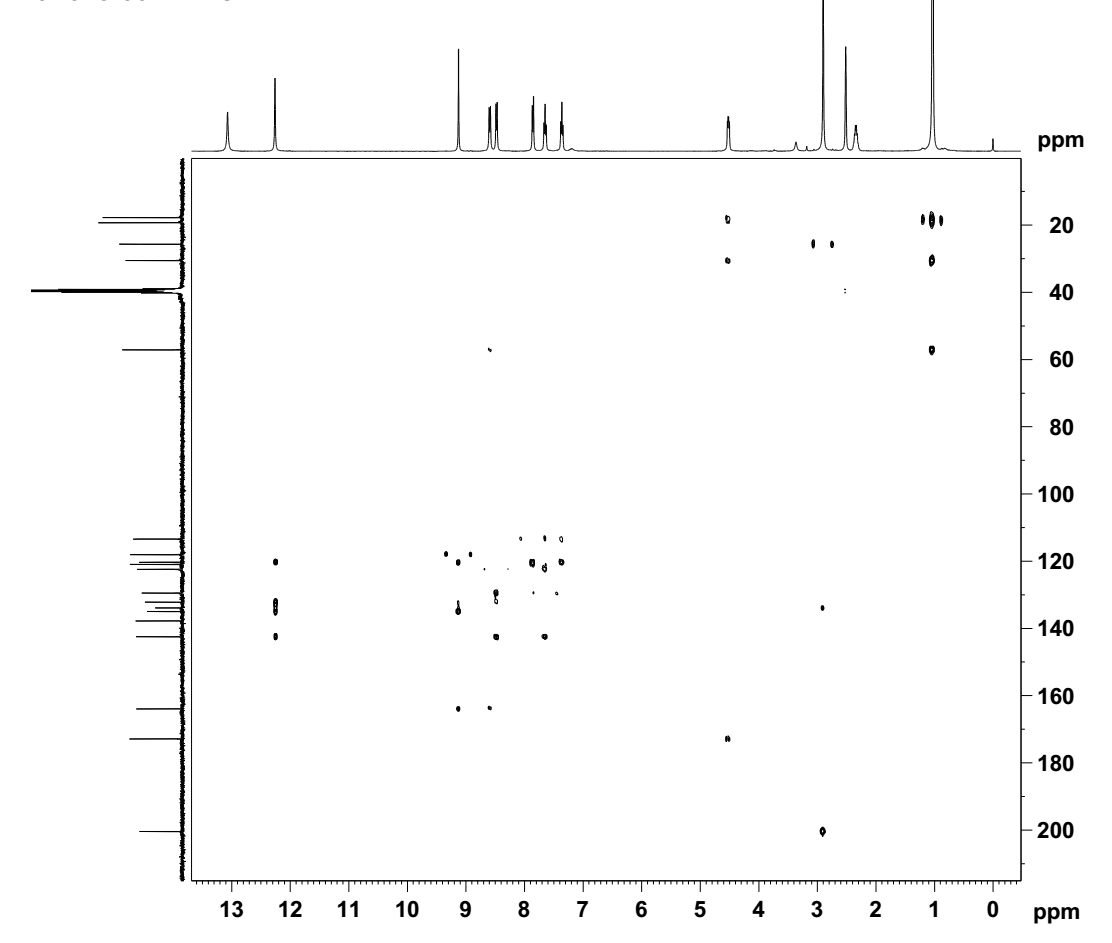


## Figure S19. HMBC spectrum of 3 in DMSO-*d*_6_.


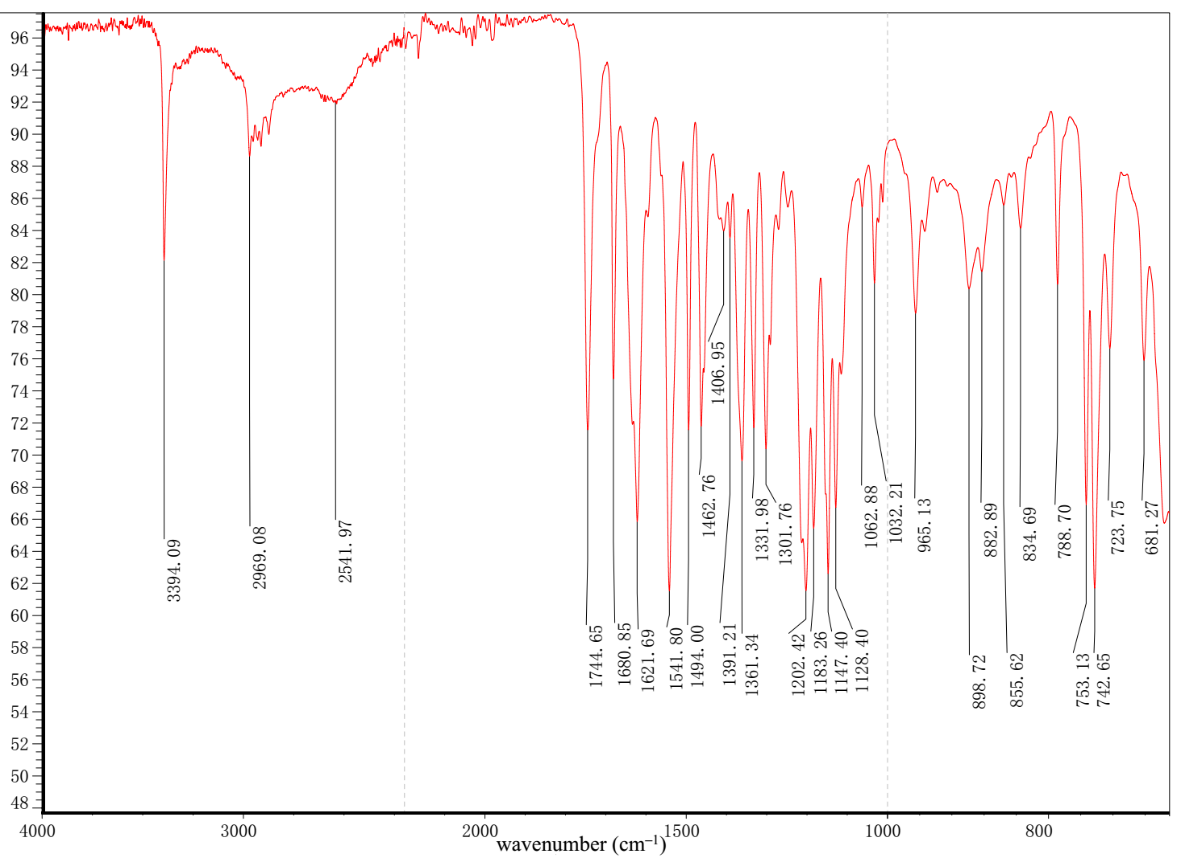


## Figure S20. IR spectrum of 3.


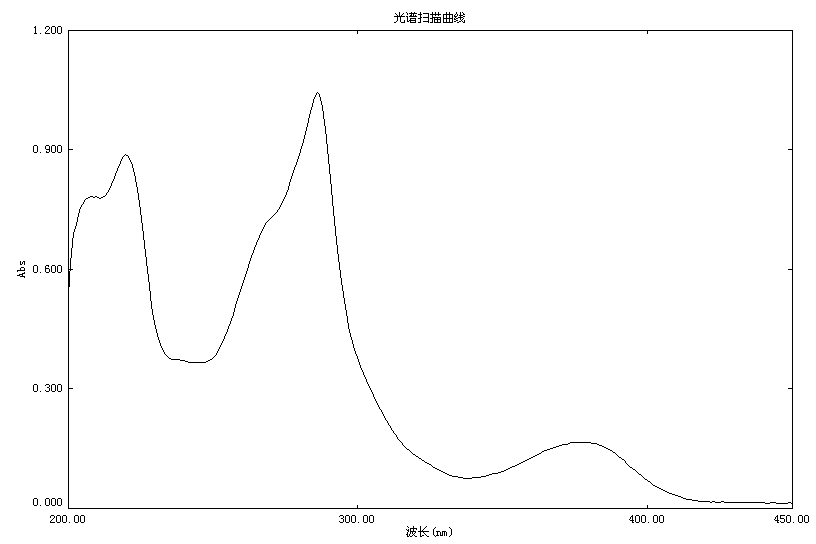


wavelength (nm)

##

## Figure S21. UV spectrum of 3 in MeOH.


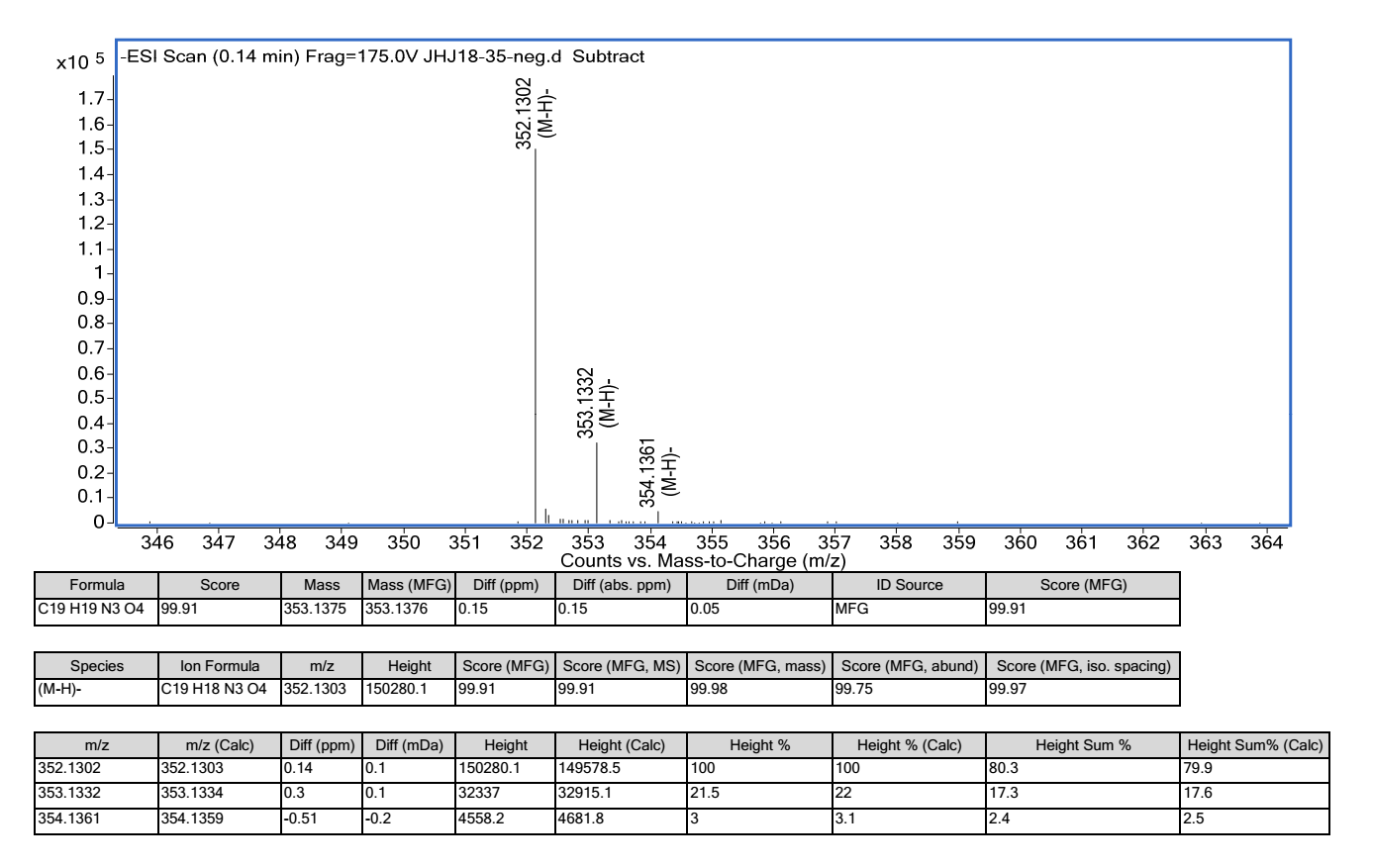


## Figure S22. HRESIMS of 3.


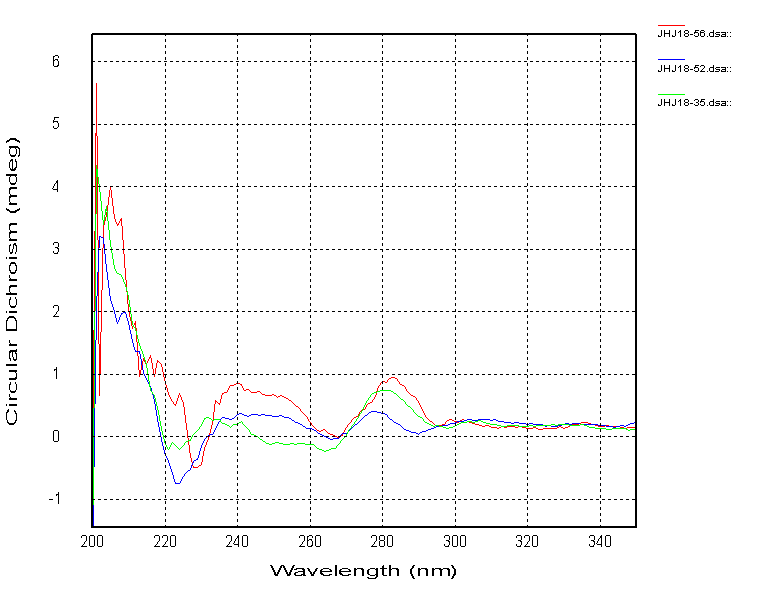


compound **7**

compound **3**

compound **2**

## Figure S23. Experimental ECD spectra of 2, 3 and 7.


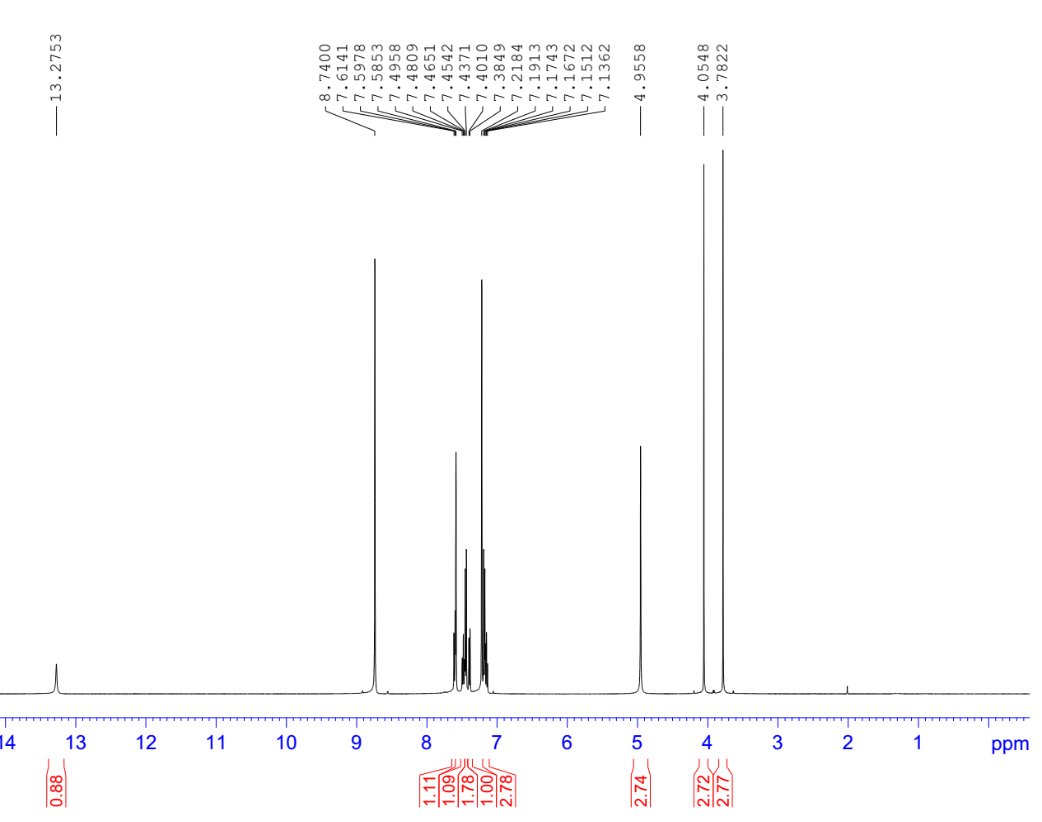


## Figure S24. ^1^H NMR spectrum (500 MHz) of 4 in Py-*d*_5_.


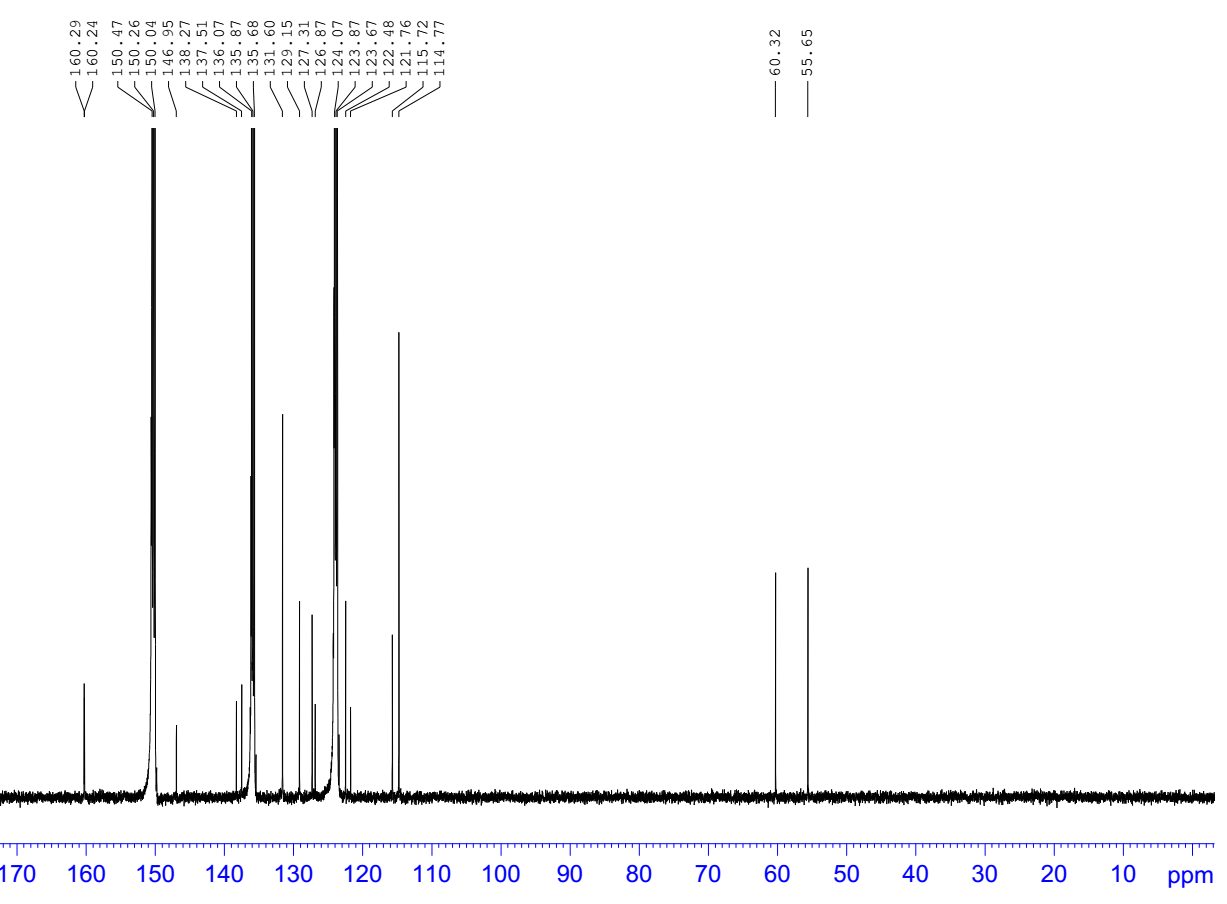


## Figure S25. ^13^C NMR spectrum (125 MHz) of 4 in Py-*d*_5_.


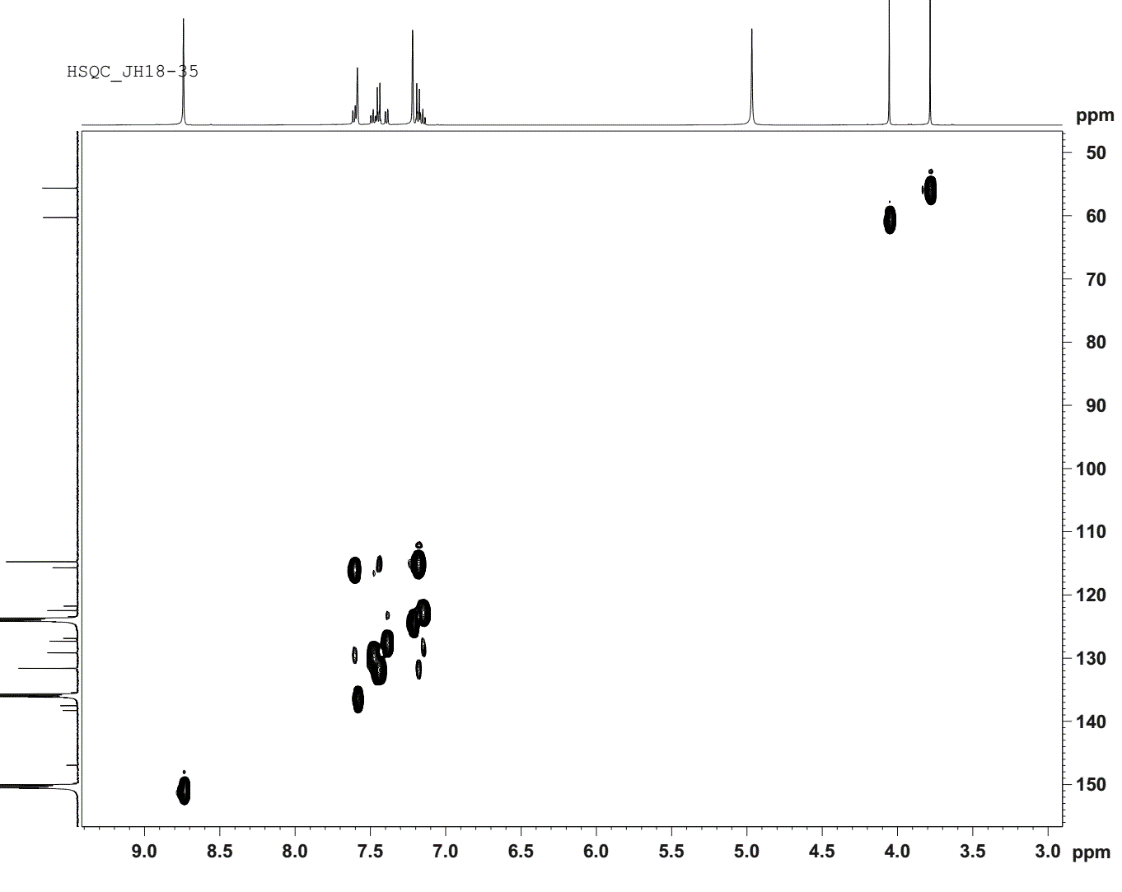


## Figure S26. HSQC spectrum of 4 in Py-*d*_5_.


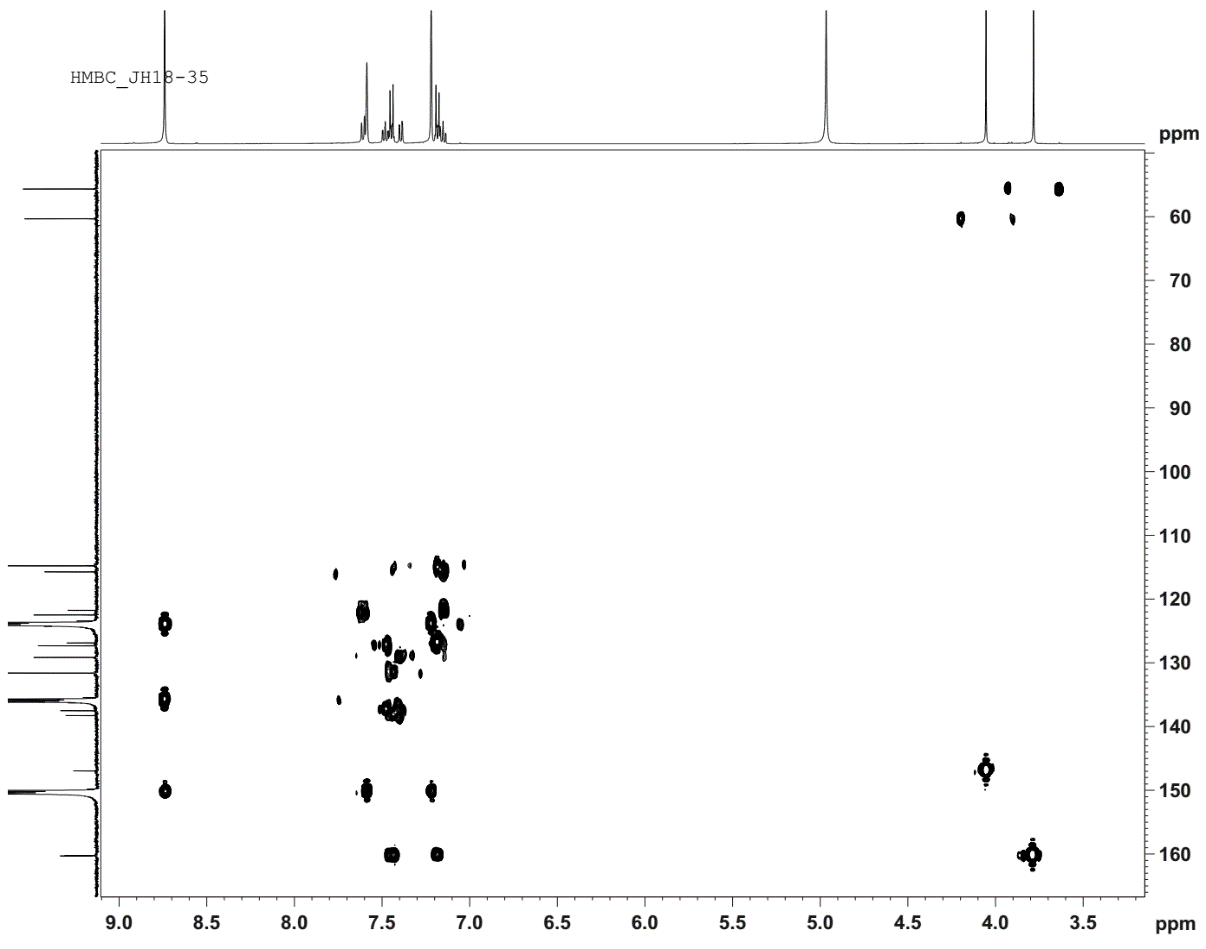


## Figure S27. HMBC spectrum of 4 in Py-*d*_5_.


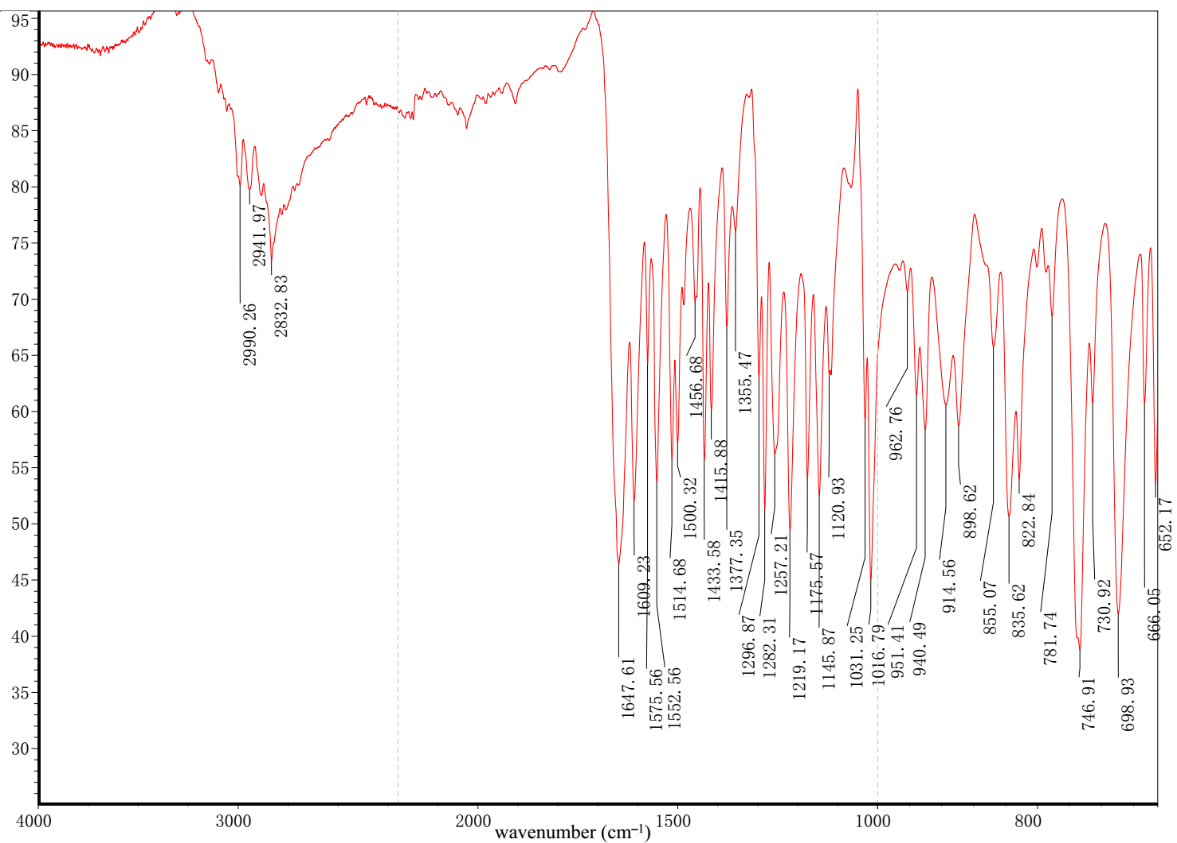


## Figure S28. IR spectrum of 4.


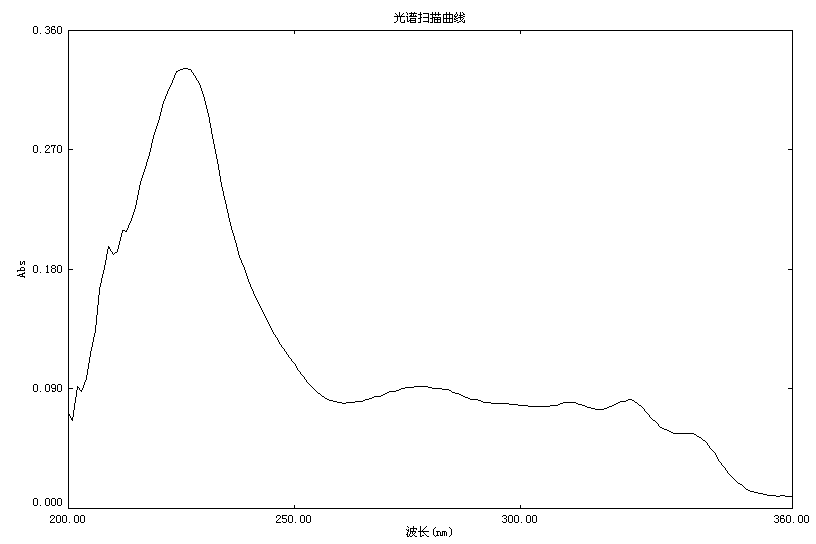


wavelength (nm)

## Figure S29. UV spectrum of 4 in MeOH.


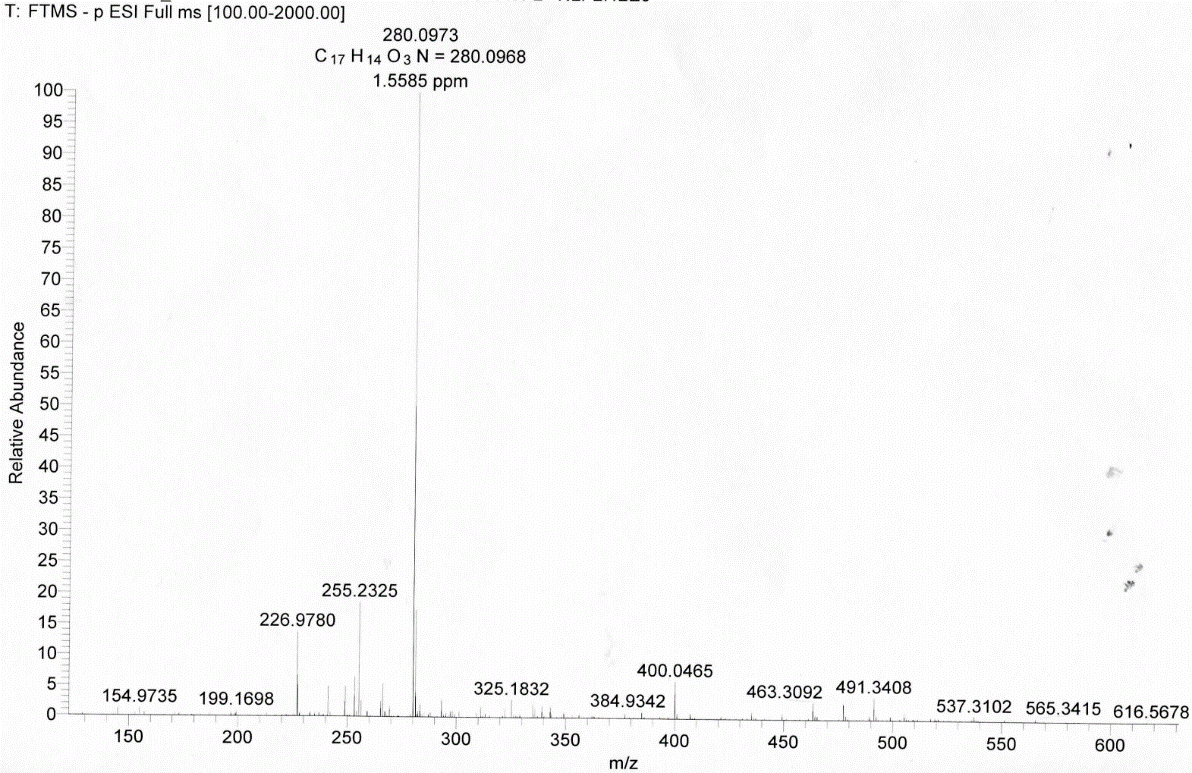


## Figure S30. HRESIMS of 4.


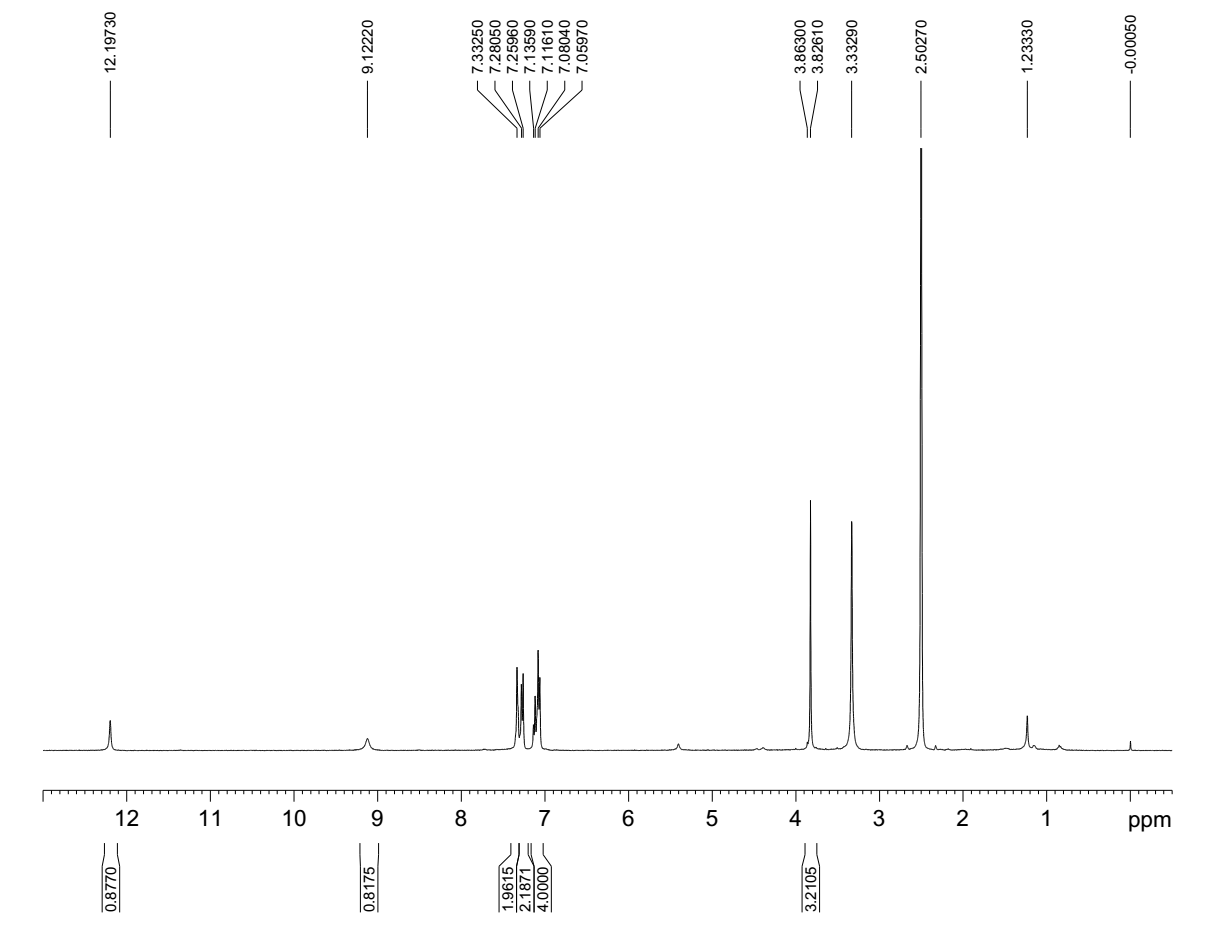


## Figure S31. ^1^H NMR spectrum (400 MHz) of 5 in DMSO-*d*_6_.


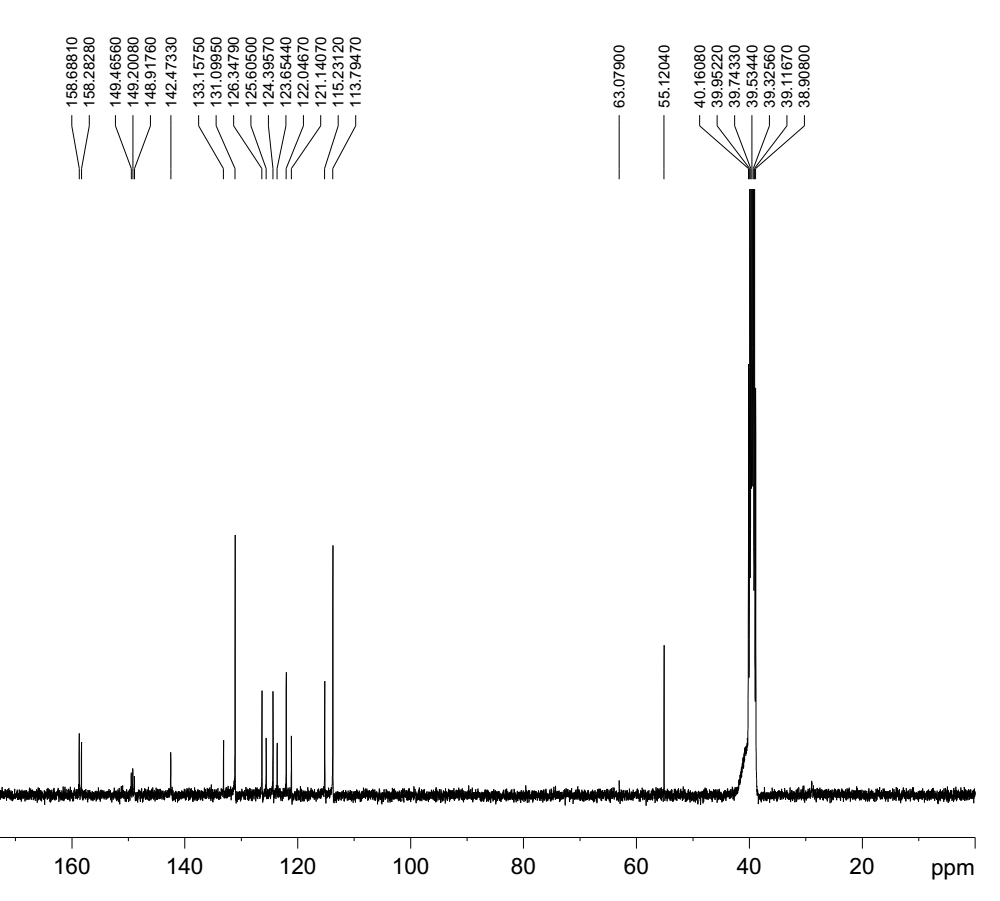


## Figure S32. ^13^C NMR spectrum (100 MHz) of 5 in DMSO-*d*_6_.


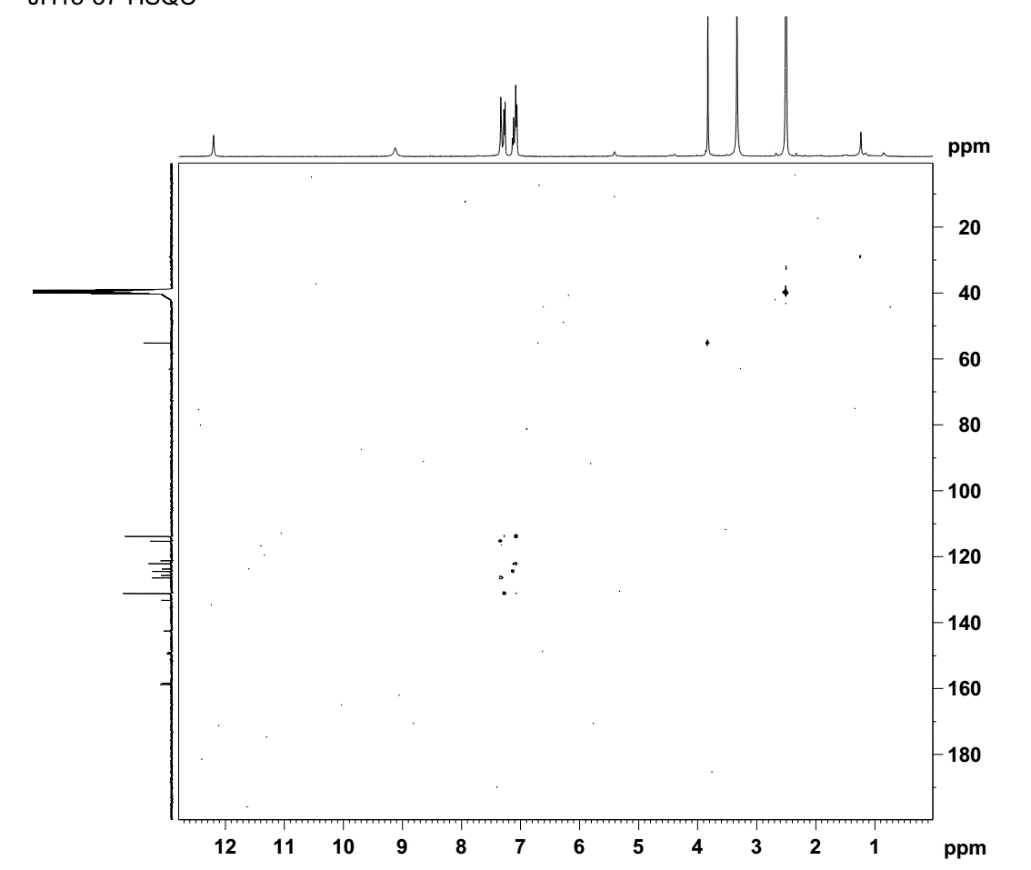


## Figure S33. HSQC spectrum of 5 in DMSO-*d*_6_.


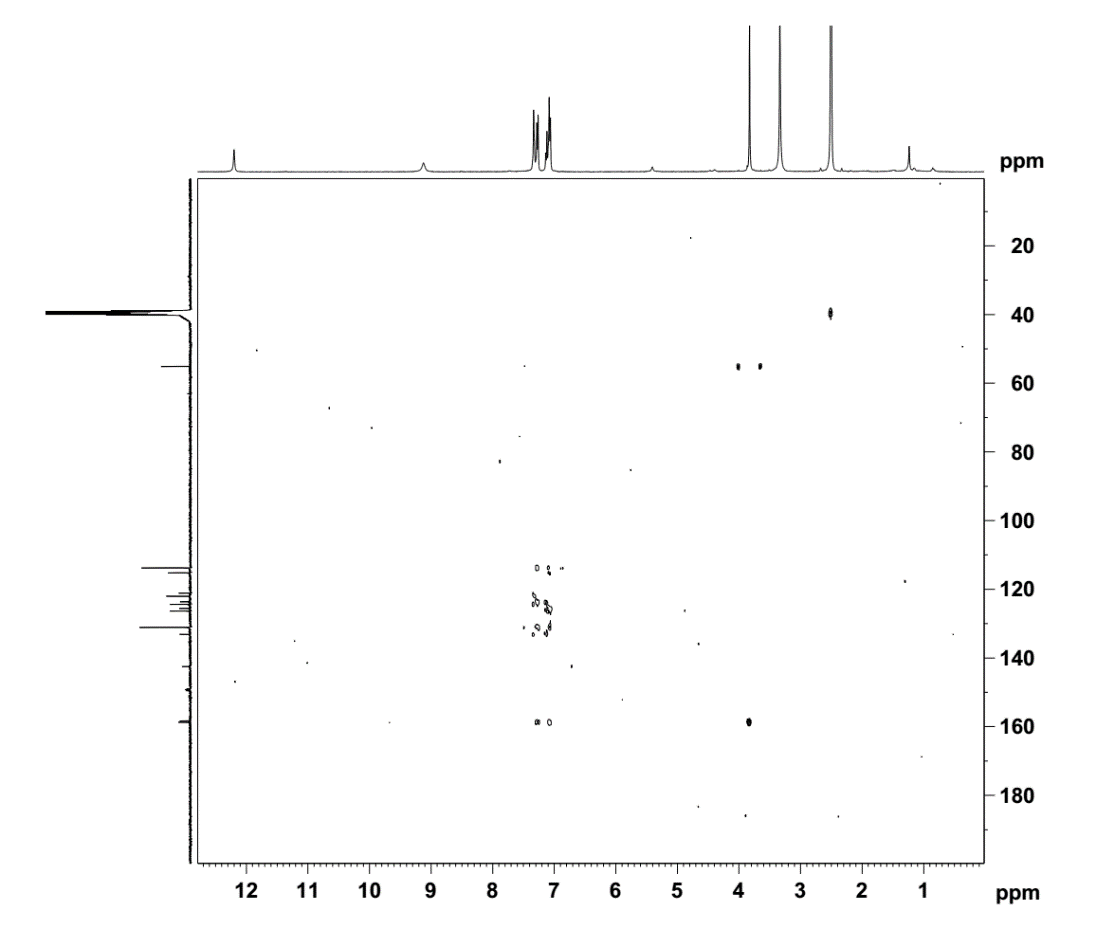


## Figure S34. HMBC spectrum of 5 in DMSO-*d*_6_.


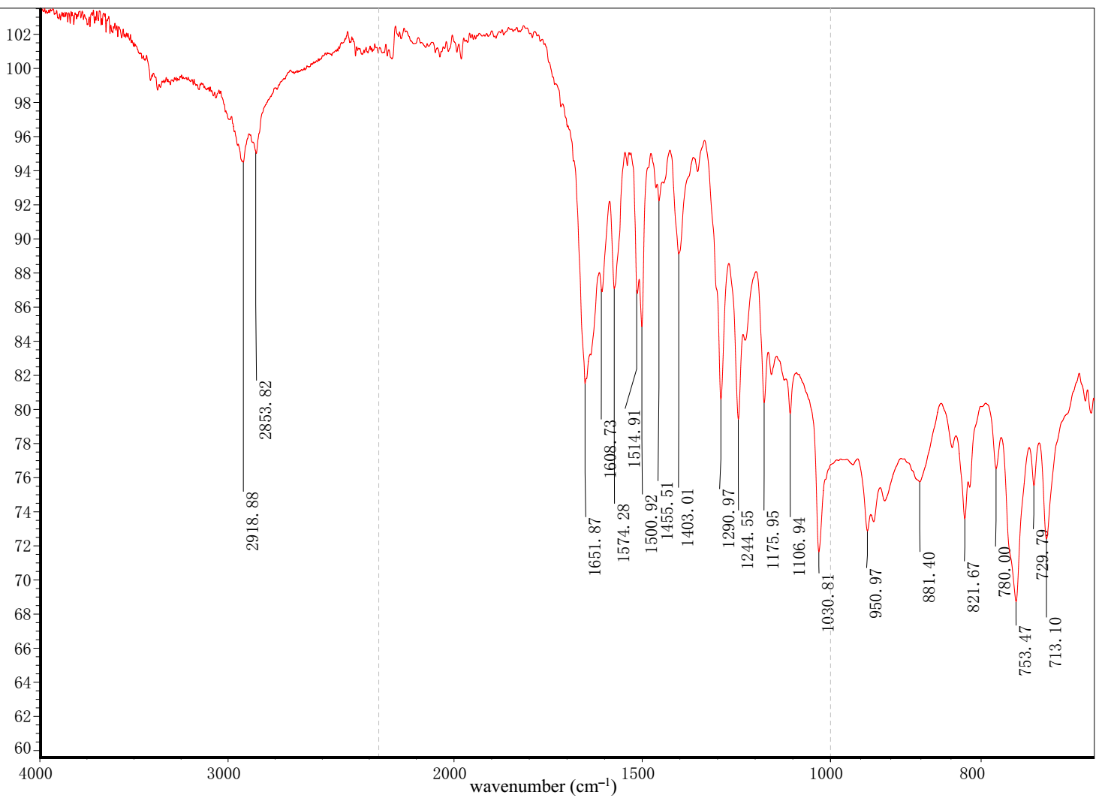


## Figure S35. IR spectrum of 5.


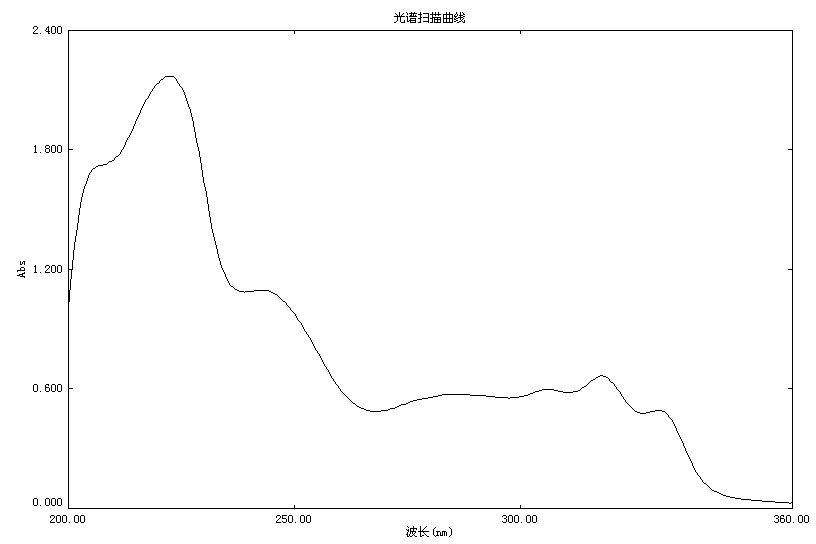


wavelength (nm)

## Figure S36. UV spectrum of 5 in MeOH.


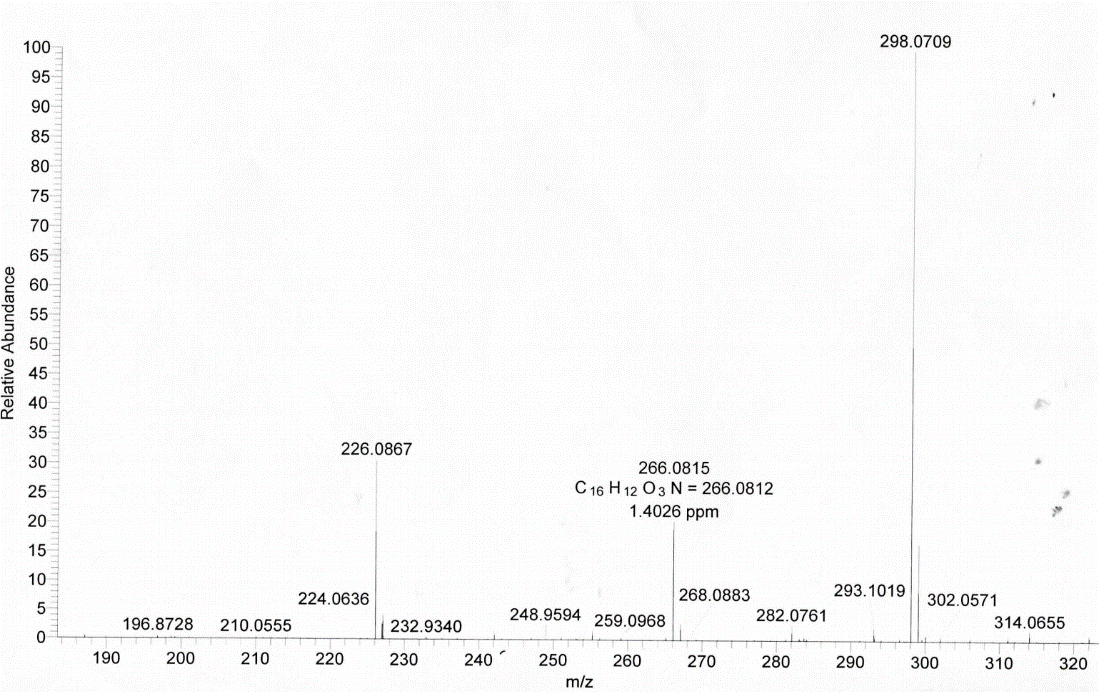


## Figure S37. HRESIMS of 5.

## Attachment S1. Supporting information for the calculated ECD spectra of compounds 1.

# Computational methods

## Conformational analysis

Conformational analysis was initially performed using Confab [1] with systematic search at MMFF94 force field for the *S* configuration of compound **1** (**Figure AS1**). Room-temperature equilibrium populations were calculated according to Boltzmann distribution law (1) and those with populations lower than 1% were filtered. The energies and populations of dominative conformers were provided in **Table AS1**.


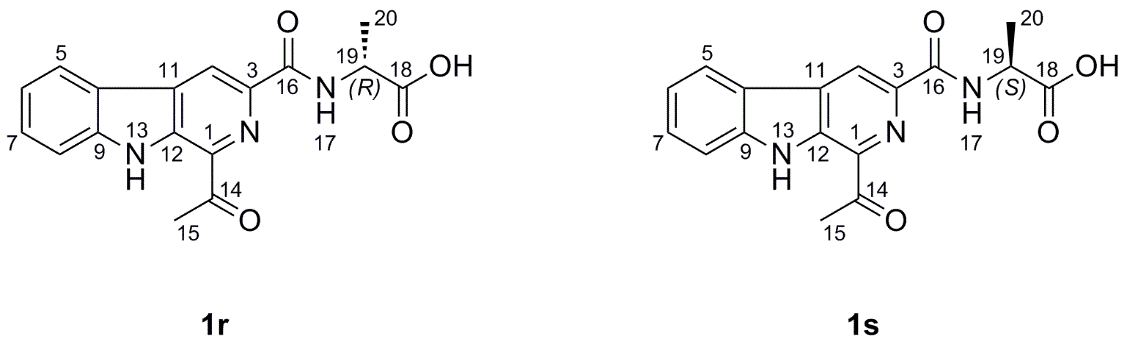


**Figure AS1** Chemical structure of compound **1**.

$\frac{N_{i}}{N}=\frac{g_{i}e^{-\frac{E_{i}}{k_{\text{B}}T}}}{\sum g_{i}e^{-\frac{E_{i}}{k_{\text{B}}T}}}$ *(1)*

where $N_{i}$is the number of conformer i with energy $E_{i}$and degeneracy $g_{i}$ at temperature *T*, and *k*_B_ is Boltzmann constant.

## ECD calculation

The theoretical calculations were carried out using Gaussian 09 ^[2]^. At first, conformers were optimized at PM6 and HF/6-31G(d) and filtered by Boltzmann-based populations. The remaining structure was finally optimized at B3LYP/6-31G(d) . Vibrational frequency analysis confirmed the stable structure. The ECD calculation was conducted at BP86/6-311G(d,p) using Time-dependent Density functional theory (TD-DFT) in methanol using the IEFPCM model. Rotatory strengths for a total of 30 excited states were calculated. The ECD spectrum was simulated in SpecDis ^[3]^ by overlapping Gaussian functions for each transition according to (2).

$\Delta\varepsilon(E)=\frac{1}{2.297\times10^{-39}}\times\frac{1}{\sqrt{2\pi\sigma}}\sum_{i}^{A} \Delta E_{i}R_{i}e^{-\left(\frac{E-E_{i}}{2\sigma}\right)^{2}}$ *(2)*

where *σ* represents the width of the band at 1/*e* height, and $\Delta E_{i}$ and $R_{i}$ are the excitation energies and rotatory strengths for transition *i*, respectively.

The *σ* and UV-shift values were 0.22 eV and -60 nm, respectively. The spectrum of configuration R is derived directly from mirror inversion of that of configuration S.

## References

1. O'Boyle, N.M., Vandermeersch, T., Flynn, C.J., Maguire, A.R. and Hutchison, G.R. (2011). Confab-systematic generation of diverse low-energy conformers. *J. Cheminformatics*, 3, 8–16.
2. Frisch, M.J., Trucks, G.W., Schlegel, H.B., Scuseria, G.E., Robb, M.A., Cheeseman, J.R., ea al. (2009). Gaussian 09 Revision D.01. Gaussian Inc., Wallingford CT.
3. Bruhn, T., Schaumlöffel, A., Hemberger, Y. and Bringmann, G. (2013). Specdis: quantifying the comparison of calculated and experimental electronic circular dichroism spectra. *Chirality* 25, 243–249.

# Energies and Coordinates


## Energies at MMFF94 force field

A systematic conformational search was performed by the Confab program at MMFF94 force field. Conformers for each configuration were obtained with filtration by RMSD threshold of 0.5 Å.

**Table AS1** Energies of configuration **1s** at MMFF94 force field.

| Compound | Conformer | Energy (kcal/mol) | Population (%) |
| --- | --- | --- | --- |
| 1s | 1 | 61.07 | 35.55 |
| 1s | 2 | 61.38 | 21.06 |
| 1s | 3 | 61.68 | 12.68 |
| 1s | 4 | 61.85 | 9.56 |
| 1s | 5 | 62.04 | 6.90 |
| 1s | 6 | 62.08 | 6.46 |
| 1s | 7 | 62.77 | 2.02 |
| 1s | 8 | 62.91 | 1.60 |
| 1s | 9 | 62.95 | 1.49 |

## Energies at B3LYP theory level

The structure of **1s** was optimized at B3LYP/6-31G(d) in gas phase.


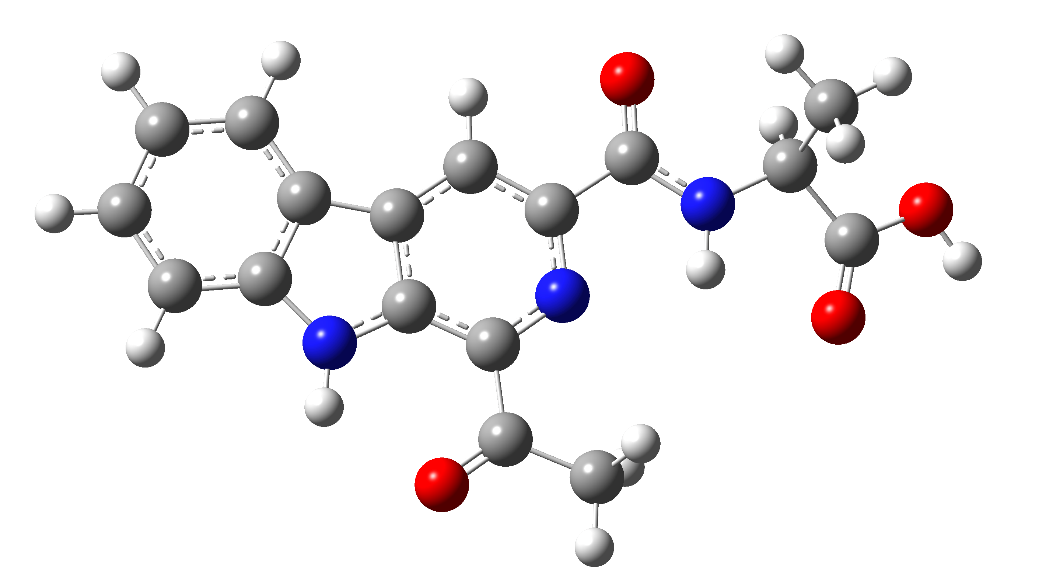


Conformer: **1s**

SCF Done: E(RB3LYP) = -1122.391943 A.U.

E = -704311.57 kcal/mol.

## Coordinates at B3LYP theory level

**Table AS2** Standard orientations of conformer **1s** at B3LYP/6-31G(d) level in gas phase.

| Center | Atomic | Atomic |  | Coordinates (Angstroms) | | |
| --- | --- | --- | --- | --- | --- | --- |
| Number | Number | Type |  | X | Y | Z |
| 1 | 7 | 0 |  | -0.528121 | 0.747750 | 0.069580 |
| 2 | 7 | 0 |  | 3.128239 | 1.257339 | 0.030140 |
| 3 | 1 | 0 |  | 3.313000 | 2.251399 | 0.069180 |
| 4 | 7 | 0 |  | -2.904281 | -0.530350 | 0.069240 |
| 5 | 1 | 0 |  | -2.777511 | 0.475600 | 0.062110 |
| 6 | 8 | 0 |  | -4.864741 | 1.238950 | -0.476010 |
| 7 | 8 | 0 |  | -6.416951 | -0.379220 | -0.698160 |
| 8 | 1 | 0 |  | -6.947711 | 0.407840 | -0.924380 |
| 9 | 8 | 0 |  | -1.865271 | -2.556030 | -0.082100 |
| 10 | 8 | 0 |  | 1.620090 | 3.570559 | 0.152500 |
| 11 | 6 | 0 |  | -5.173431 | 0.067540 | -0.420340 |
| 12 | 6 | 0 |  | -4.246211 | -1.065970 | -0.013510 |
| 13 | 1 | 0 |  | -4.275001 | -1.833630 | -0.796900 |
| 14 | 6 | 0 |  | -4.711431 | -1.716380 | 1.306240 |
| 15 | 1 | 0 |  | -4.015221 | -2.519560 | 1.557840 |
| 16 | 1 | 0 |  | -4.715021 | -0.981380 | 2.118000 |
| 17 | 1 | 0 |  | -5.716261 | -2.134500 | 1.197900 |
| 18 | 6 | 0 |  | -1.809171 | -1.327560 | -0.006460 |
| 19 | 6 | 0 |  | -0.494411 | -0.592950 | 0.010440 |
| 20 | 6 | 0 |  | 0.688959 | -1.344231 | -0.041270 |
| 21 | 1 | 0 |  | 0.617199 | -2.425341 | -0.087990 |
| 22 | 6 | 0 |  | 1.896039 | -0.655801 | -0.031000 |
| 23 | 6 | 0 |  | 1.851999 | 0.767009 | 0.030490 |
| 24 | 6 | 0 |  | 0.618289 | 1.441739 | 0.080270 |
| 25 | 6 | 0 |  | 0.571170 | 2.927769 | 0.145430 |
| 26 | 6 | 0 |  | -0.777990 | 3.604540 | 0.204500 |
| 27 | 1 | 0 |  | -0.633320 | 4.686140 | 0.227040 |
| 28 | 1 | 0 |  | -1.328980 | 3.281430 | 1.094630 |
| 29 | 1 | 0 |  | -1.391390 | 3.322370 | -0.657770 |
| 30 | 6 | 0 |  | 3.298439 | -1.017831 | -0.069160 |
| 31 | 6 | 0 |  | 4.028289 | 0.200909 | -0.029480 |
| 32 | 6 | 0 |  | 5.424389 | 0.222719 | -0.051350 |
| 33 | 1 | 0 |  | 5.974739 | 1.158479 | -0.020940 |
| 34 | 6 | 0 |  | 6.084599 | -1.001621 | -0.113300 |
| 35 | 1 | 0 |  | 7.170829 | -1.016102 | -0.131410 |
| 36 | 6 | 0 |  | 5.378539 | -2.218391 | -0.152910 |
| 37 | 1 | 0 |  | 5.928399 | -3.153561 | -0.201000 |
| 38 | 6 | 0 |  | 3.987889 | -2.234991 | -0.131360 |
| 39 | 1 | 0 |  | 3.444639 | -3.175421 | -0.162250 |

# Experimental and calculated ECD spectra

**Figure AS2** Calculated ECD spectra of compounds **1** were compared with the experimental.
