# Supplementary material for: Alkaloid diversity expansion of a talent fungus Penicillium raistrichii through OSMAC-based cultivation
Source: Front Microbiol. 2023 Nov 9;14:1279140. doi: 10.3389/fmicb.2023.1279140 (PMC10665910; doi:10.3389/fmicb.2023.1279140)

checkCIF/PLATON report

You have not supplied any structure factors. As a result the full set of tests cannot be run.

THIS REPORT IS FOR GUIDANCE ONLY. IF USED AS PART OF A REVIEW PROCEDURE FOR PUBLICATION, IT SHOULD NOT REPLACE THE EXPERTISE OF AN EXPERIENCED CRYSTALLOGRAPHIC REFEREE.

No syntax errors found.   CIF dictionary   Interpreting this report

Datablock: 160425a

```
Bond precision:  C-C = 0.0086 A          Wavelength=1.54178

Cell:           a=15.8834(11)    b=6.8525(7)       c=18.4959(17)
                alpha=90         beta=90.565(8)    gamma=90
Temperature:    293 K

                Calculated                 Reported
Volume          2013.0(3)                  2013.0(3)
Space group     C 2                        C2
Hall group      C 2y                       ?
Moiety formula  C21 H23 N3 O4              ?
Sum formula     C21 H23 N3 O4              C21 H23 N3 O4
Mr              381.42                     381.42
Dx,g cm-3       1.259                      1.259
Z               4                          4
Mu (mm-1)       0.723                      0.723
F000            808.0                      808.0
F000'           810.54
h,k,lmax        18,8,21                    18,8,21
Nref            3513[ 1918]                2354
Tmin,Tmax       0.771,0.924                0.737,0.925
Tmin'           0.722

Correction method= # Reported T Limits: Tmin=0.737 Tmax=0.925
AbsCorr = MULTI-SCAN

Data completeness= 1.23/0.67      Theta(max)= 66.040

R(reflections)= 0.0633( 1353)     wR2(reflections)= 0.1650( 2354)

S = 1.046                   Npar= 259
```

The following ALERTS were generated. Each ALERT has the format
test-name_ALERT_alert-type_alert-level.
Click on the hyperlinks for more details of the test.

Alert level C

```
STRVA01_ALERT_4_C           Flack test results are meaningless.
           From the CIF: _refine_ls_abs_structure_Flack    0.000
           From the CIF: _refine_ls_abs_structure_Flack_su    0.900
PLAT220_ALERT_2_C Non-Solvent  Resd 1  C   Ueq(max)/Ueq(min) Range        4.8 Ratio
PLAT222_ALERT_3_C Non-Solv.  Resd 1  H   Uiso(max)/Uiso(min) Range        5.5 Ratio
PLAT234_ALERT_4_C Large Hirshfeld Difference C3       --C6       .       0.22 Ang.
PLAT242_ALERT_2_C Low    'MainMol' Ueq as Compared to Neighbors of        C1 Check
PLAT242_ALERT_2_C Low    'MainMol' Ueq as Compared to Neighbors of        C3 Check
PLAT242_ALERT_2_C Low    'MainMol' Ueq as Compared to Neighbors of        C4 Check
PLAT340_ALERT_3_C Low Bond Precision on  C-C Bonds ...............    0.00863 Ang.
```

Alert level G

```
PLAT005_ALERT_5_G No Embedded Refinement Details Found  in the CIF     Please Do !
PLAT007_ALERT_5_G Number of Unrefined Donor-H Atoms ..............          2 Report
PLAT032_ALERT_4_G Std. Uncertainty on Flack Parameter Value High .      0.900 Report
PLAT093_ALERT_1_G No s.u.'s on H-positions, Refinement Reported as      mixed Check
PLAT199_ALERT_1_G Reported _cell_measurement_temperature ..... (K)        293 Check
PLAT200_ALERT_1_G Reported   _diffrn_ambient_temperature ..... (K)        293 Check
PLAT791_ALERT_4_G Model has Chirality at C2          (Chiral SPGR)          S Verify
PLAT791_ALERT_4_G Model has Chirality at C3          (Chiral SPGR)          S Verify
PLAT850_ALERT_4_G Check Flack Parameter Exact Value 0.00 and s.u.        0.90 Check
PLAT899_ALERT_4_G SHELXL97   is Deprecated and Succeeded by SHELXL       2018 Note
```

0 **ALERT level A** = Most likely a serious problem - resolve or explain
0 **ALERT level B** = A potentially serious problem, consider carefully
8 **ALERT level C** = Check. Ensure it is not caused by an omission or oversight
10 **ALERT level G** = General information/check it is not something unexpected

3 ALERT type 1 CIF construction/syntax error, inconsistent or missing data
4 ALERT type 2 Indicator that the structure model may be wrong or deficient
2 ALERT type 3 Indicator that the structure quality may be low
7 ALERT type 4 Improvement, methodology, query or suggestion
2 ALERT type 5 Informative message, check

It is advisable to attempt to resolve as many as possible of the alerts in all categories. Often the minor alerts point to easily fixed oversights, errors and omissions in your CIF or refinement strategy, so attention to these fine details can be worthwhile. In order to resolve some of the more serious problems it may be necessary to carry out additional measurements or structure refinements. However, the purpose of your study may justify the reported deviations and the more serious of these should normally be commented upon in the discussion or experimental section of a paper or in the "special_details" fields of the CIF. checkCIF was carefully designed to identify outliers and unusual parameters, but every test has its limitations and alerts that are not important in a particular case may appear. Conversely, the absence of alerts does not guarantee there are no aspects of the results needing attention. It is up to the individual to critically assess their own results and, if necessary, seek expert advice.

Publication of your CIF in IUCr journals

A basic structural check has been run on your CIF. These basic checks will be run on all CIFs submitted for publication in IUCr journals (*Acta Crystallographica*, *Journal of Applied Crystallography*, *Journal of Synchrotron Radiation*); however, if you intend to submit to *Acta Crystallographica Section C* or *E* or *IUCrData*, you should make sure that full publication checks are run on the final version of your CIF prior to submission.

Publication of your CIF in other journals

Please refer to the *Notes for Authors* of the relevant journal for any special instructions relating to CIF submission.

PLATON version of 03/05/2019; check.def file version of 29/04/2019

Datablock 160425a - ellipsoid plot

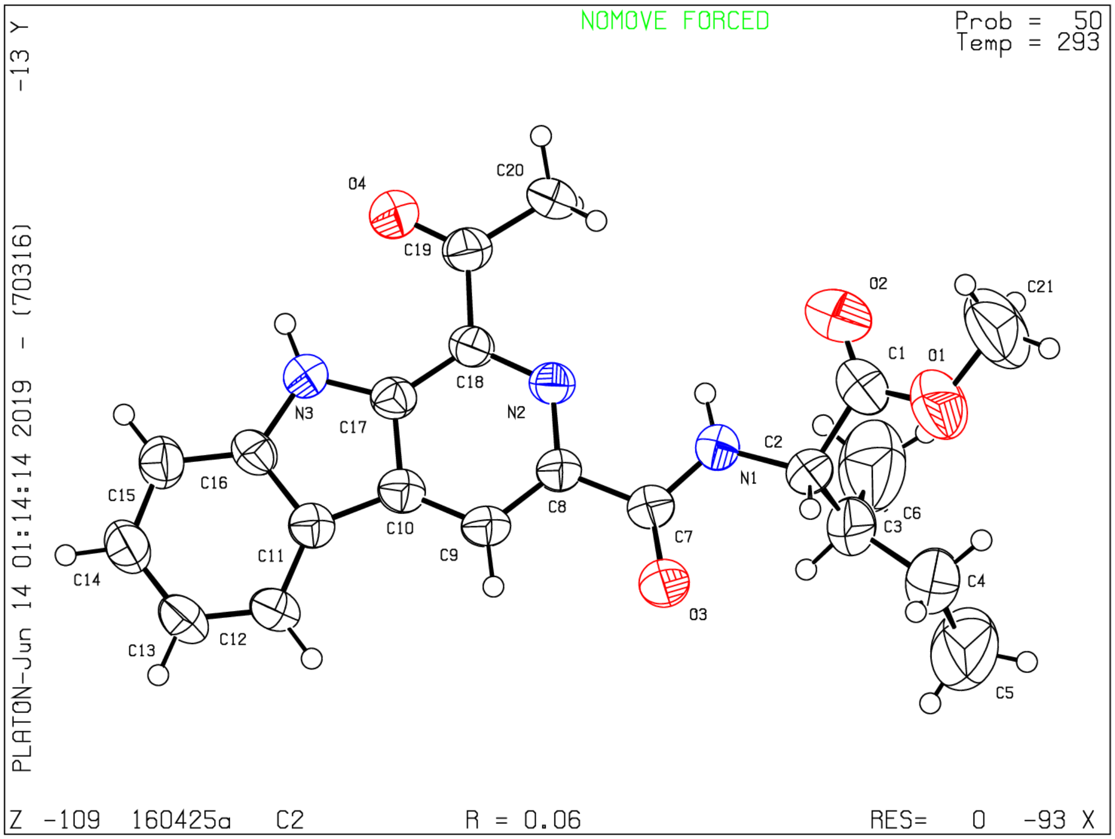

Supplement: Supplementary file 2 [file Data_Sheet_2.zip › Checkcif 24679/(NO FCF)/compound 2 checkcif.pdf]
