# Supplementary material for: Alkaloid diversity expansion of a talent fungus Penicillium raistrichii through OSMAC-based cultivation
Source: Front Microbiol. 2023 Nov 9;14:1279140. doi: 10.3389/fmicb.2023.1279140 (PMC10665910; doi:10.3389/fmicb.2023.1279140)

checkCIF/PLATON report

You have not supplied any structure factors. As a result the full set of tests cannot be run.

THIS REPORT IS FOR GUIDANCE ONLY. IF USED AS PART OF A REVIEW PROCEDURE FOR PUBLICATION, IT SHOULD NOT REPLACE THE EXPERTISE OF AN EXPERIENCED CRYSTALLOGRAPHIC REFEREE.

No syntax errors found. CIF dictionary Interpreting this report

Datablock: 150520g

```
Bond precision:  C-C = 0.0042 A          Wavelength=0.71073

Cell:             a=15.8992(14)      b=8.5824(8)     c=23.758(2)
                  alpha=90           beta=90         gamma=90
Temperature:      298 K

                Calculated                Reported
Volume          3241.9(5)                 3241.8(5)
Space group     P b c a                   Pbca
Hall group      -P 2ac 2ab                ?
Moiety formula  C18 H16 N2 O3             ?
Sum formula     C18 H16 N2 O3             C18 H16 N2 O3
Mr              308.33                    308.33
Dx,g cm-3       1.263                     1.263
Z               8                         8
Mu (mm-1)       0.087                     0.087
F000            1296.0                    1296.0
F000'           1296.60
h,k,lmax        18,10,28                  18,10,28
Nref            2863                      2861
Tmin,Tmax       0.972,0.980               0.963,0.980
Tmin'           0.963

Correction method= # Reported T Limits: Tmin=0.963 Tmax=0.980
AbsCorr = MULTI-SCAN

Data completeness= 0.999          Theta(max)= 25.020

R(reflections)= 0.0516( 1462)     wR2(reflections)= 0.1146( 2861)

S = 1.014                    Npar= 210
```

The following ALERTS were generated. Each ALERT has the format
test-name_ALERT_alert-type_alert-level.
Click on the hyperlinks for more details of the test.

Alert level C

```
PLAT220_ALERT_2_C NonSolvent   Resd 1  C   Ueq(max)/Ueq(min) Range        3.1 Ratio
PLAT230_ALERT_2_C Hirshfeld Test Diff for    O3       --C17      .        6.0 s.u.
PLAT242_ALERT_2_C Low    'MainMol' Ueq as Compared to Neighbors of        C14 Check
PLAT340_ALERT_3_C Low Bond Precision on  C-C Bonds ...............    0.00419 Ang.
PLAT601_ALERT_2_C Unit Cell Contains Solvent Accessible VOIDS of .         35 Ang**3
```

Alert level G

```
PLAT005_ALERT_5_G No Embedded Refinement Details Found  in the CIF     Please Do !
PLAT007_ALERT_5_G Number of Unrefined Donor-H Atoms ..............          1 Report
PLAT066_ALERT_1_G Predicted and Reported Tmin&Tmax Range Identical          ? Check
PLAT093_ALERT_1_G No s.u.'s on H-positions, Refinement Reported as      mixed Check
PLAT899_ALERT_4_G SHELXL97  is Deprecated and Succeeded by SHELXL/       2018 Note
```

0 **ALERT level A** = Most likely a serious problem - resolve or explain
0 **ALERT level B** = A potentially serious problem, consider carefully
5 **ALERT level C** = Check. Ensure it is not caused by an omission or oversight
5 **ALERT level G** = General information/check it is not something unexpected

2 ALERT type 1 CIF construction/syntax error, inconsistent or missing data
4 ALERT type 2 Indicator that the structure model may be wrong or deficient
1 ALERT type 3 Indicator that the structure quality may be low
1 ALERT type 4 Improvement, methodology, query or suggestion
2 ALERT type 5 Informative message, check

It is advisable to attempt to resolve as many as possible of the alerts in all categories. Often the minor alerts point to easily fixed oversights, errors and omissions in your CIF or refinement strategy, so attention to these fine details can be worthwhile. In order to resolve some of the more serious problems it may be necessary to carry out additional measurements or structure refinements. However, the purpose of your study may justify the reported deviations and the more serious of these should normally be commented upon in the discussion or experimental section of a paper or in the "special_details" fields of the CIF. checkCIF was carefully designed to identify outliers and unusual parameters, but every test has its limitations and alerts that are not important in a particular case may appear. Conversely, the absence of alerts does not guarantee there are no aspects of the results needing attention. It is up to the individual to critically assess their own results and, if necessary, seek expert advice.

Publication of your CIF in IUCr journals

A basic structural check has been run on your CIF. These basic checks will be run on all CIFs submitted for publication in IUCr journals (*Acta Crystallographica*, *Journal of Applied Crystallography*, *Journal of Synchrotron Radiation*); however, if you intend to submit to *Acta Crystallographica Section C* or *E* or *IUCrData*, you should make sure that full publication checks are run on the final version of your CIF prior to submission.

Publication of your CIF in other journals

Please refer to the *Notes for Authors* of the relevant journal for any special instructions relating to CIF submission.

PLATON version of 16/07/2020; check.def file version of 12/07/2020

Datablock 150520g - ellipsoid plot

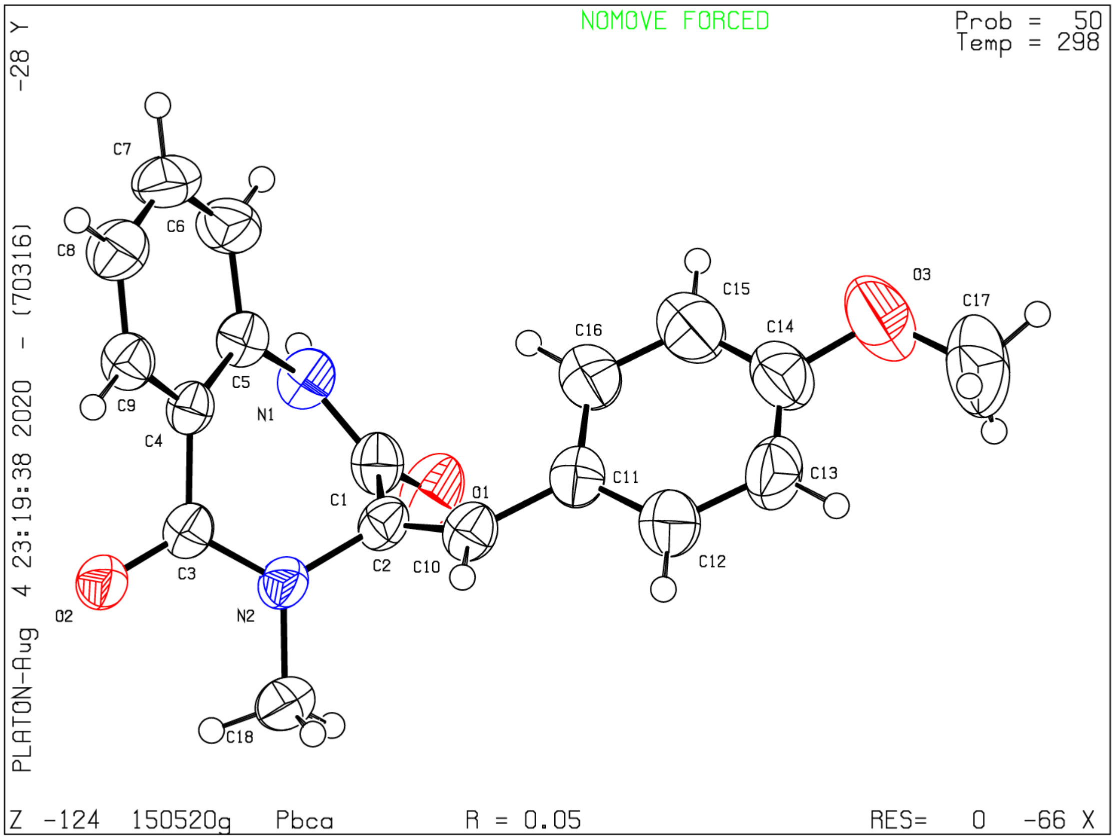

Supplement: Supplementary file 2 [file Data_Sheet_2.zip › Checkcif 24679/(NO FCF)/compound 9 checkcif.pdf]
